# In Vitro Assessment of the Role of p53 on Chemotherapy Treatments in Neuroblastoma Cell Lines

**DOI:** 10.3390/ph14111184

**Published:** 2021-11-19

**Authors:** Idoia Blanco-Luquin, Paula Lázcoz, Jon Celay, Javier S. Castresana, Ignacio J. Encío

**Affiliations:** 1 Department of Health Sciences, Public University of Navarra (UPNA), IdiSNA (Navarra Institute for Health Research), 31008 Pamplona, Spain; idoia.blanco.luquin@navarra.es (I.B.-L.); plazcozr@educacion.navarra.es (P.L.); icelayle@unav.es (J.C.); 2 Department of Biochemistry and Genetics, University of Navarra School of Sciences, 31008 Pamplona, Spain

**Keywords:** neuroblastoma, chemotherapy, retinoic acid, p53, p14, MDM2, MYCN

## Abstract

Neuroblastoma is the most frequent malignant extracranial solid tumor of infancy. The overall objective of this work consists of determining the presence of alterations in the p53/MDM2/p14ARF signaling pathway in neuroblastoma cell lines and deciphering their possible relationship with resistance to known antineoplastic drugs and to differentiation agents. Firstly, we characterized 10 neuroblastoma cell lines for alterations at the p53/MDM2/p14ARF signaling pathway by analysis of TP53 point mutations, MYCN and MDM2 amplification, and p14ARF methylation, homozygous deletions, and expression. Secondly, we chose SK-N-FI (mutated at TP53) and SK-N-Be(2) (wild-type TP53) cell lines, treated them with chemotherapeutic agents (doxorubicin, etoposide, cisplatin, and melphalan) and with two isomers of retinoic acid (RA): (9-*cis* and all-*trans*). Finally, we analyzed the distribution of the cell cycle, the induction of apoptosis, and the expression levels of p53, p21, and Bcl-2 in those two cell lines. P14ARF did not present promoter methylation, homozygous deletions, and protein expression in any of the 10 neuroblastoma cell lines. One TP53 point mutation was detected in the SK-N-FI cell line. MYCN amplification was frequent, while most cell lines did not present MDM2 amplification. Treatment of SK-N-FI and SK-N-Be(2) cells with doxorubicin, etoposide, cisplatin, and melphalan increased apoptosis and blocked the cycle in G2/M, while retinoic acid isomers induced apoptosis and decreased the percentage of cells in S phase in TP53 mutated SK-N-FI cells, but not in TP53 wild-type SK-N-Be(2) cells. Treatment with cisplatin, melphalan, or 9-*cis* RA decreased p53 expression levels in SK-N-FI cells but not in SK-N-Be (2). The expression of p21 was not modified in either of the two cell lines. Bcl-2 levels were reduced only in SK-N-FI cells after treatment with cisplatin. However, treatments with doxorubicin, etoposide, or 9-*cis*-RA did not modify the levels of this protein in either of the two cell lines. In conclusion, TP53 mutated SK-N-FI cells respond better to the retinoic isomers than TP53 wild-type SK-N-Be(2) cells. Although these are in vitro results, it seems that deciphering the molecular alterations of the p53/MDM2/p14ARF signaling pathway prior to treating patients of neuroblastoma might be useful for standardizing therapies with the aim of improving survival.

## 1. Introduction

Treatment of neuroblastoma is multidisciplinary and includes surgery, chemotherapy, radiation therapy, and immunotherapy [1,2]. Chemotherapy, used for the first time in neuroblastoma in 1946 [3], is indicated in disseminated neuroblastoma and in unresectable localized neuroblastoma, in which it is administered with the aim of facilitating subsequent resection of the tumor. In tumors with metastatic spread, chemotherapy is aimed at controlling metastases and reducing the primary tumor to facilitate its subsequent resection. In these cases, the response to induction chemotherapy is consolidated with myeloablative chemotherapy and autologous bone marrow progenitor cell transplantation [1,2].

Chemotherapeutic protocols in neuroblastoma combine, among others, DNA alkylating agents (cisplatin, melphalan) and topoisomerase inhibitors (etoposide and doxorubicin). They cause cellular toxicity, eventually leading to DNA double strand breakage, resulting in p53 activation, cell cycle arrest, and induction of apoptosis cell death [4,5].

The benefit of relatively unconventional biological antitumor agents was first demonstrated in a landmark clinical trial by the Children’s Cancer Group in which the administration of the differentiating agent retinoic acid following recovery from chemotherapy high-dose treatment and stem cell transplantation improved survival for patients with high-risk neuroblastoma [6]. Complete cure requires not only removal of the immediately identifiable tumor, but also eradication of minimal residual disease that remains after high-dose therapy. In this framework, the administration of retinoic acid has become standard therapy [7,8,9]. Retinoic acid exerts growth inhibitory and differentiating effects through its interaction with nuclear retinoic acid receptors (RARs), which regulate the expression of multiple target genes [10].

TP53 gene mutations are the most common genetic alterations observed in sporadic human cancers [11], occurring in approximately 30–50% of all human tumors, indicating that loss of p53 activity provides a substantial advantage for cell transformation and uncontrolled proliferation [12]. However, a striking feature of neuroblastoma is its low frequency (<2%) of TP53 mutations at diagnosis [13,14,15,16]. However, there is considerable evidence to suggest that TP53 mutations could be acquired during chemotherapy and malignant progression of neuroblastoma [14]. Accordingly, an increased frequency of TP53 mutations is observed in neuroblastoma cell lines that show drug resistance and in those established in relapse [13,14]. More than 80% of TP53 mutations in human cancers are concentrated between exons 5 and 8 of the gene (codons 110 to 307). TP53 neuroblastoma mutations occur as missense (81.82%), nonsense (13.64%), and silent mutations (4.54%) (https://p53.iarc.fr, accessed on 8 July 2021) (Table S1). Unlike other tumor suppressors, the functional inactivation of *TP53* mainly relies on point mutations with a high incidence in certain hot spot residues. In neuroblastoma, these include, above all, codon 135 and less frequently codon 176 and others (International Agency for Research on Cancer (http://www–p53.iarc.fr, accessed on 8 July 2021) (Table S1). Most of these point mutations are located in the central DNA-binding domain of the p53 protein and thus affect the specific binding of p53 to promoters of its target genes. However, depending on the mutated residue, p53 mutated proteins could have a residual transactivation capacity and still be able to induce the expression of certain transcripts [17]. Finally, another potential mechanism by which certain tumor cells with wild-type p53 gene might develop resistance to chemotherapeutic agents could be the alteration of nuclear import of p53 protein by RA [18].

The MDM2 oncoprotein [19] is a critical negative regulator of p53 stability and activity. It is well established that the functional interaction between p53 and MDM2 is absolutely essential. Under normal physiological conditions, p53 levels are very low due to its proteasomal degradation dependent on MDM2 [20]. Exposure of cells to harmful stimuli results in post-translational stabilization of p53 through a wide array of different modification mechanisms of the protein, including acetylation, ribosylation, O-glycosylation, SUMOylation, and NEDDylation. This suppresses the binding of p53 to MDM2 and leads to accumulation and increased transcriptional activity of p53 [20]. In addition to inducing the expression of target genes involved in cell cycle arrest, DNA damage repair, senescence, and apoptosis, p53 increases MDM2 expression, limiting the duration and intensity of a non-lethal stress response, ensuring the degradation of p53 itself under stress-free physiological conditions [21].

A central negative regulator of MDM2 is the tumor suppressor protein p14ARF, which can bind to MDM2 and inhibit its ubiquitin ligase activity, preventing the degradation of p53 [14,22] and thus maintaining p53 in an active state [15]. p14ARF is a product of the alternative reading frame of the CDKN2A locus on chromosome 9p21. The p14ARF protein acts as a key sensor for hyperproliferative signals generated by activated oncogenes (such as MYC or RAS) and involves both pathways, dependent or independent of p53, to protect cells from malignant transformation [14].

Amplification of the MYCN oncogene plays a central role in the pathophysiology and clinical behavior of high-risk neuroblastoma and is associated with an increased vascular index and poor prognosis [23]. However, abnormal MYCN expression also potently sensitizes neuroblastoma cells to drug- and stress-induced apoptosis, and therefore needs to be accompanied by another collateral impairment of the cell death program to provide a tumor selective advantage [24]. This counteracting action of MYCN’s intrinsic apoptosis-sensitizing effect may be triggered by increased MDM2 activity (despite the absence of gene amplification) [13,24], so that MYCN-directed MDM2 expression could constitutively weaken the pathway of p53 in neuroblastoma cells with amplified MYCN. Certainly, Tran et al. [24] demonstrate that MDM2 promotes MYCN translation and MYCN-dependent proliferation via a p53-independent mechanism. Quite the contrary, MYCN induces MDM2 expression in neuroblastoma cells. All of which reveals a high specific collaboration between MDM2 and MYCN oncoproteins in this tumor.

Identifying alterations in the p53/MDM2/p14ARF pathway would make it possible to obtain an early molecular diagnosis and a prognostic prediction, and would facilitate the administration of the most appropriate treatments for each neuroblastoma patient. For this reason, the global objective of this work consists of determining the presence of alterations in the p53/MDM2/p14ARF signaling pathway in neuroblastoma cell lines and deciphering their possible relationship with resistance to known antineoplastic drugs and to differentiation agents. For that purpose, we have studied TP53 mutations, MYCN and MDM2 amplification, p14ARF methylation, expression, and homozygous deletions, and have analyzed the effects of chemotherapeutic agents (doxorubicin, etoposide, cisplatin, and melphalan) and of two isomers of retinoic acid (9-*cis*-RA and all*-trans*-RA (*at*-RA)) on the distribution of the cell cycle, the induction of apoptosis, and the expression levels of p53, p21, and Bcl-2 in neuroblastoma cell lines.

## 2. Results

### 2.1. Methylation of the P14ARF Promoter

The degree of methylation of the promoter of the P14ARF gene, located on chromosome 9p21–22, was studied in 12 cell lines (SK–N–DZ, SK–N–SH, SK–N–Be(2), SK–N–FI, BE(2)C, IMR–32, Kelly, SIMA, SH–SY5Y, MHH–NB–11, SK–N–MC, and MC–IXC) using MSP (methylation-specific PCR) and MCA-Meth (melting curve analysis–methylation assay).

#### 2.1.1. MSP (Methylation-Specific PCR)

**Figure 1:**
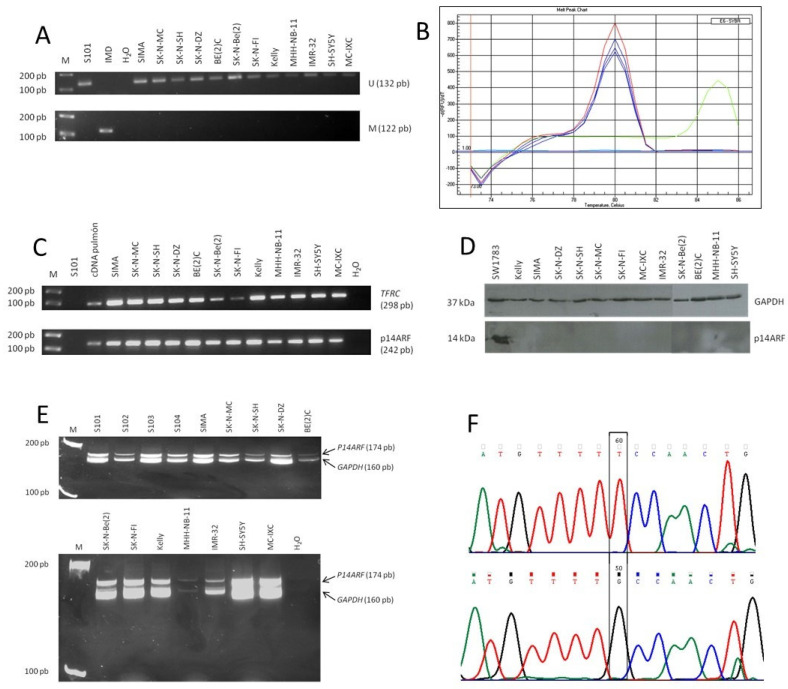
Detection of p14ARF and TP53 alterations. (**A**) Analysis of the degree of methylation of the P14ARF promoter: 2.5% agarose gels stained with ethidium bromide (0.1 µg/mL). M: molecular weight marker (1 Kb Plus DNA Ladder, Invitrogen). S101: peripheral blood from a healthy donor. IMD: in vitro methylated DNA; (**B**) MCA-Meth assay of the P14ARF gene in the SIMA cell line. The denaturation curve of the PCR product obtained with DNA from blood cells (unmethylated control) is in red color, while that obtained with in vitro methylated DNA (methylation control) is in green color; those of the SIMA cell line appear in blue color (*n* = 3); (**C**) RT-PCR expression analysis of the p14ARF gene. The PCR products were analyzed on 2.5% agarose gels stained with ethidium bromide (0.1 µg/mL). M: molecular weight marker; S101: genomic DNA; lung cDNA: cDNA from normal lung tissue (normal expression positive control); (**D**) Western Blot analysis of p14ARF. Proteins were separated by SDS-PAGE in 15% gradient polyacrylamide gels. Immunoblots were visualized by using enhanced chemiluminescence. Protein values were normalized regarding the amount of GAPDH; (**E**) differential PCR for detection of P14ARF homozygous deletions: 15% polyacrylamide gels (19:1) stained with ethidium bromide (0.2 µg/mL). M: molecular weight marker; S101, S102, S103, S104: genomic DNA extracted from peripheral blood of healthy donors (negative control); (**F**) sequencing reaction of the 5′ end of exon 5 of the TP53 gene in the neuroblastoma cell line SK-N-FI (above) and in the control DNA (below). Chromatogram section containing the mutated base in the SK-N-FI cell line.

After treatment with sodium bisulfite, the U primer pair, complementary to the unmethylated DNA sequence, produced amplification of the DNAs (Figure 1A). However, with the M primer pair, complementary to the methylated DNA template, the product of the amplification reaction was not observed in any of the cell lines. This result indicates that the promoter of the P14ARF gene is not methylated in any of the cell lines analyzed.

#### 2.1.2. MCA–Meth (Melting Curve Analysis–Methylation Assay)

Figure 1B shows the result obtained with the SIMA cell line as an example. The product of the PCR reactions carried out on DNA extracted from this cell line (triplicate, blue lines) is denatured at the same temperature as the DNA extracted from the peripheral blood of healthy donors (negative control, red line). However, the PCR product obtained from in vitro methylated DNA (positive control, green line) is denatured at a higher temperature, because after treatment with sodium bisulfite it maintains the CG base pairs. Similar results were obtained in the rest of the cell lines analyzed (Figure S1), which confirms that the promoter of the P14ARF gene is not methylated in any of them.

### 2.2. p14ARF Expression

#### 2.2.1. Detection of mRNA by Semi-Quantitative RT-PCR

Obtained results on the expression of the P14ARF gene in the 12 cell lines (SK–N–DZ, SK–N–SH, SK–N–Be(2), SK–N–FI, BE(2)C, IMR–32, Kelly, SIMA, SH–SY5Y, MHH–NB–11, SK–N–MC, and MC–IXC) are shown in Figure 1C. No loss of expression of p14ARF was observed in any of the samples.

#### 2.2.2. Protein Detection by Western Blot

p14ARF protein expression in the 12 cell lines was analyzed by Western Blot. Results are shown in Figure 1D, in which the SW1783 glioma line was included as a positive control. The presence of p14ARF could only be detected in the glioma cell line used as a control. To correct for possible differences in the amount of protein loaded between samples, the membrane was washed and retested with a specific antibody against GAPDH. The results obtained confirm the absence of p14ARF in the analyzed samples.

### 2.3. p14ARF Homozygous Deletions

Results are shown in Figure 1E. Two bands were obtained in all cell lines analyzed, of which the largest one corresponds to the P14ARF gene and the smallest to the internal control GAPDH. In none of the cases was the intensity ratio between both bands less than 1/3 of the mean ratio obtained in the non-doubtful samples, which makes it possible to rule out the presence of P14ARF homozygous deletions in these samples.

### 2.4. TP53 Gene Mutations

The PCR products obtained by amplifying exons 5 to 9 of TP53 were then sequenced in the 12 cell lines (SK–N–DZ, SK–N–SH, SK–N–Be(2), SK–N–FI, BE(2)C, IMR–32, Kelly, SIMA, SH–SY5Y, MHH–NB–11, SK–N–MC, and MC–IXC) looking for possible mutations. Sequence analysis showed a point mutation at the 5′ end of exon 5 in neuroblastoma SK-N-FI cells (Figure 1F). It corresponds to the change of guanine for thymine at codon 135. The mutation was confirmed by sequencing both strands. The substitution of the amino acid cysteine (Cys, TGC triplet) for phenylalanine (Phe, TTC triplet) implies a non-conservative mutation within the DNA-binding motif of this protein that could affect its activity.

### 2.5. MYCN and MDM2 Gene Amplification by FISH

FISH (fluorescent in situ hybridization) was carried out to quantify the copy number of the MYCN and MDM2 genes in cell lines SK-N-DZ, SK-N-SH, SK-N-Be(2), SK-N-FI, BE(2)C, IMR-32, Kelly, SIMA, SH-SY5Y, MHH-NB-11, SK-N-MC, and MC-IXC. In the case of MYCN, the RP11-744F11 bacterial artificial chromosome (BAC), labeled with Spectrum Red, was used as a probe; the specific probe for the centromere of chromosome 2 (α2) was labeled with Spectrum Green and used as a reference. To determine the amplification status of the MDM2 gene, the RP11-77H17 BAC was used as a specific probe for MDM2, labeled with Spectrum Red; α12 centromeric probe labeled with Spectrum Green was used as a reference. Results are shown in Figure S2 and are summarized in Table 1.

In the case of the MDM2 gene, no amplification of the gene was observed in any of the 12 cell lines analyzed by FISH (Table 1). Thus, the count of the major clone in the SK–N–MC, SK–N–FI, IMR–32, Kelly, SK–N–Be(2), SH–SY5Y, and SK–N–SH cell lines was two green signals from α12 and two red signals from RP11-77H17; therefore, the hybridization pattern was considered normal (Figure S2 [13,14,15,16,17,18,19]). The MC-IXC cell line showed three α12 signals and two RP11-77H17 signals (3V 2R), suggesting loss of the MDM2 gene (Figure S2 [20]). Finally, the cytogenetic analysis showed a polyploid karyotype without *MDM2* amplification for the BE(2)C, SK-N-DZ, SIMA, and MHH-NB-11 cell lines (Figure S2 [21,22,23,24]). Results on the genetic profile of the neuroblastoma cell lines are shown in Table 2.

### 2.6. Cytotoxicity Assays

Next, the cytotoxicity of the compounds doxorubicin, etoposide, cisplatin, melphalan, 9-*cis*-RA, and *at*-RA was analyzed on the neuroblastoma cell lines SK-N-FI and SK-N- Be(2). These cell lines were selected based on the results obtained with them in the previous sections: mutation of TP53 in SK-N-FI, loss of chromosome 2 in SK-N-FI (aneuploidy), polyploid metaphases in SK-N-Be(2), and homogeneously staining region MYCN amplification in two unknown derived chromosomes in both cell lines. Growth inhibition after 72 h of treatment was determined by the MTT colorimetric assay. Results are represented in dose–response curves for each compound (Figure S3). As shown in the figure, the two cell lines showed different chemosensitivity with respect to cisplatin, the SK-N-Be(2) cells being more sensitive to treatment (GI50 = 4.12 µM) than SK-N-FI cells (GI50 = 29.55 µM). However, in cytotoxicity assays with the compounds doxorubicin, etoposide, melphalan, 9-*cis*-RA, and *at*-RA, no differences were detected in terms of cell survival between the two cell lines, which showed similar dose–response curves. Furthermore, it should be noted that treatment with doxorubicin, etoposide, or melphalan inhibited the cell viability of both lines in a concentration-dependent manner with GI50, TGI, and LC50 values in the micromolar order. However, this reduction in the percentage of living cells was not observed in the retinoic acid treatments, as only 9-*cis*-RA showed a slight cytostatic effect in SK-N-Be (2) cells. Therefore, these results evidenced the need to carry out additional experiments to analyze whether the effect of the treatments on cell viability was related to the induction of apoptosis or the modification in the distribution of the cell cycle.

### 2.7. Apoptosis Detection

**Figure 2:**
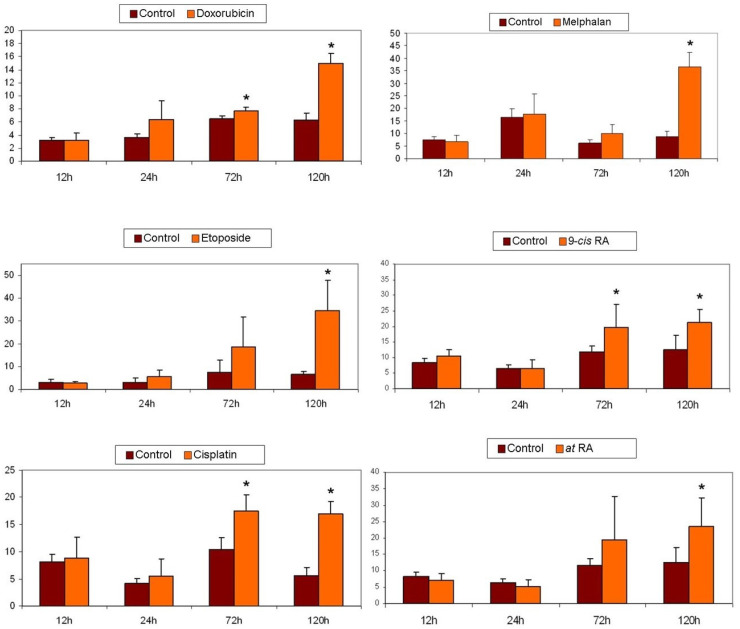
Induction of apoptosis in SK-N-FI neuroblastoma cells. Apoptosis determination was carried out by the TUNEL method. Results obtained in a representative experiment are shown. The subdiploid cells were considered apoptotic. Histograms obtained with the corresponding data from at least three independent experiments performed in duplicate. Values are expressed as mean ± SEM. * *p* < 0.05.

In order to analyze the ability of the compounds doxorubicin, etoposide, cisplatin, melphalan, 9-*cis*-RA, and *at*-RA to induce apoptosis in the neuroblastoma cell lines SK–N–FI y SK–N–Be(2), the cells were cultured in DMEM + L-Glutamax supplemented with 10% FBS, 5% non-essential amino acids, and 5 µg/mL of plasmocin. SK-N-FI cells revealed a statistically significant increase in apoptosis levels at 120 h of treatment with each of the compounds, although this increase was already noticeable at 72 h with doxorubicin, cisplatin, and 9-*cis*-RA (Figure 2). It is worth highlighting the effect of etoposide, which after 120 h of treatment multiplies almost six times the percentage of apoptotic cells in SK-N-FI cultures. It is followed by melphalan, cisplatin, and doxorubicin, in which the level of apoptosis rises, respectively, more than four, three, and two times.

**Figure 3:**
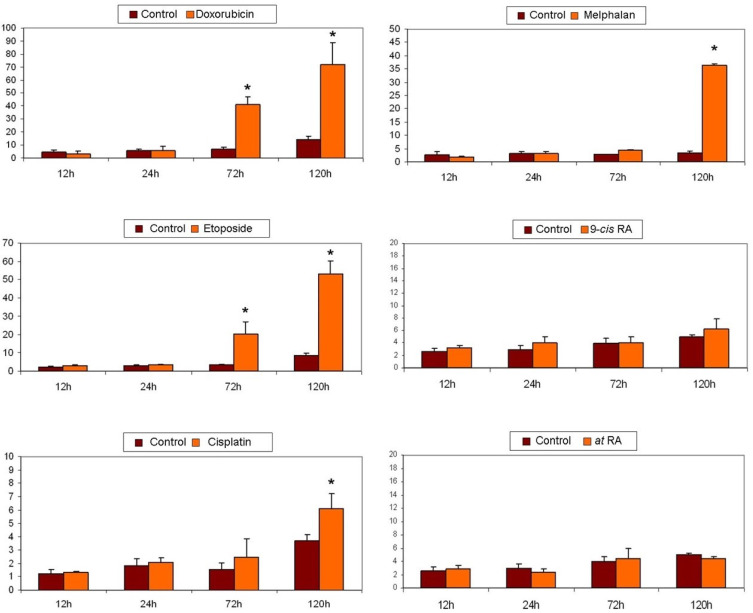
Induction of apoptosis in SK-N-Be(2) neuroblastoma cells. Apoptosis determination was carried out by the TUNEL method. Results obtained in a representative experiment are shown. The subdiploid cells were considered apoptotic. Histograms obtained with the corresponding data from at least three independent experiments performed in duplicate. Values are expressed as mean ± SEM. * *p* < 0.05.

The action of these compounds on the SK-N-Be(2) cell line was also determined (Figure 3). The chemotherapeutic agents melphalan, etoposide, and doxorubicin induced, at 120 h, an increase in the percentage of apoptotic cells in the cultures of more than 10, 6, or 5 times, respectively. In the case of doxorubicin and etoposide, significant differences were already observed in terms of the number of apoptotic cells from 72 h. Treatment with cisplatin for 120 h increased, without doubling, the level of apoptosis with respect to the control. The apoptosis induction study concluded with treatments with different isoforms of retinoic acid, which in none of the cases modified the apoptosis levels in the cultures.

In summary, with the exception of etoposide, the rest of the compounds showed a different capacity to induce apoptosis in cultures depending on the cell line. Thus, while melphalan and doxorubicin were more effective in SK-N-Be(2), cisplatin and the two isoforms of retinoic acid acted more potently in the SK-N-FI cell line, which is mutated in the TP53 gene.

### 2.8. Cell Cycle Analysis

The effect of doxorubicin, etoposide, cisplatin, melphalan, 9-*cis*-RA, and *at*-RA on the distribution of the cell cycle in SK-N-FI and SK-N-Be(2) cells was estimated by flow cytometry after 12, 24, 72, or 120 h of treatment with the compounds, at concentrations that varied between 0.2 and 1.5 µM depending on the compound used. As previously observed in the apoptosis detection studies, cell cycle distribution was more affected in SK-N-Be(2) cells than in SK-N-FI cells. In all cases except that of *at*-RA, the first cell cycle alteration observed in both cell lines was an increase in the Sub-G1 phase that, in general, became greater with time.

**Figure 4:**
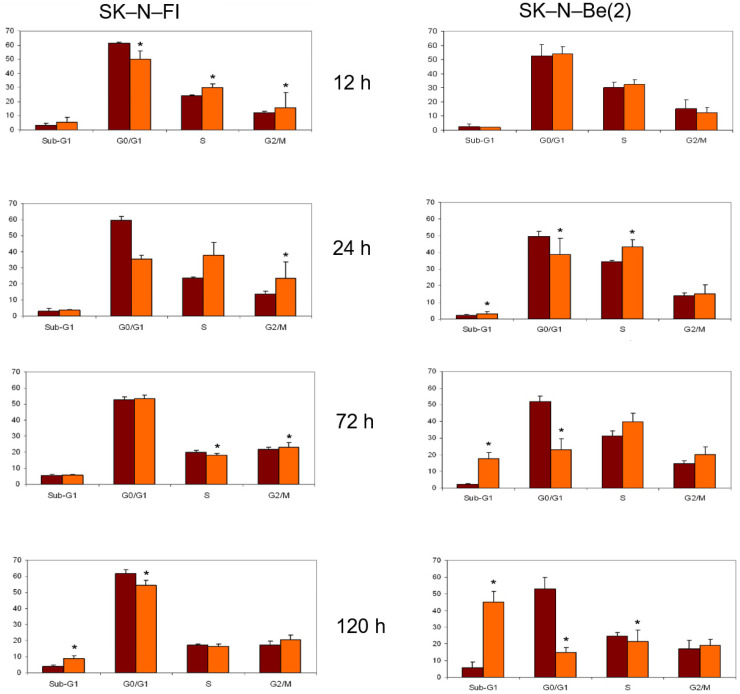
Effect of 0.2 μM doxorubicin treatment on cell cycle distribution in SK-N-FI and SK-N-Be(2) neuroblastoma cell lines. Histogram that collects the aggregated results of at least three independent experiments performed in duplicate. Results are expressed as mean ± SEM. * *p* < 0.05. Dark column: control; clear column: doxorubicin.

The treatment with doxorubicin at a concentration of 0.2 µM decreased the percentage of cells in the G0/G1 phase, having a more pronounced impact on SK-N-Be(2) cells. Furthermore, in both cases there was an increase of cells in S and G2 phases that seemed to cease at longer times (Figure 4).

**Figure 5:**
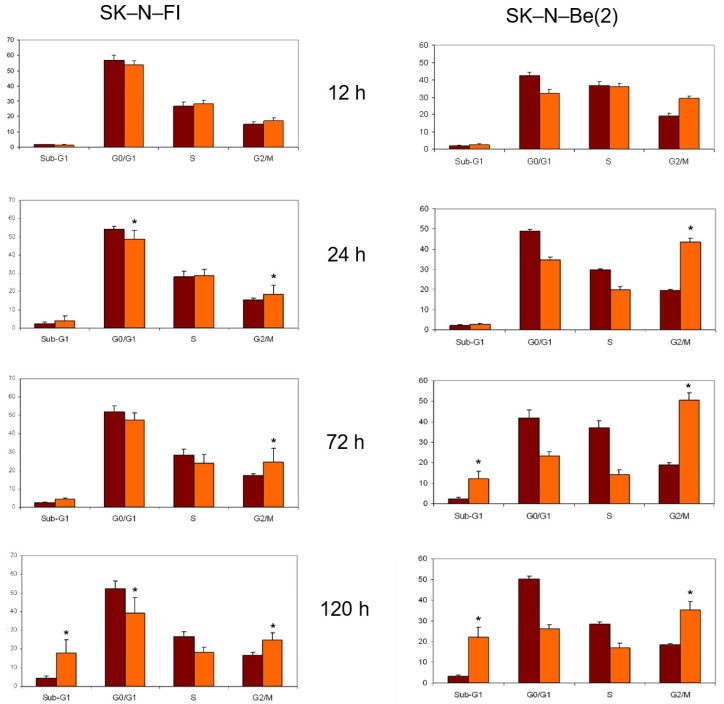
Effect of 0.5 μM etoposide treatment on cell cycle distribution in SK-N-FI and SK-N-Be(2) neuroblastoma cell lines. Histogram that collects the aggregated results of at least three independent experiments performed in duplicate. Results are expressed as mean ± SEM. * *p* < 0.05. Dark column: control; clear column: etoposide.

In relation to the study of treatment with etoposide, we again observed a decrease in the SK-N-FI and SK-N-Be(2) cell population in the G0/G1 phase as time progressed, which was seen more clearly in SK-N-Be(2) cells. We found the same effect for the percentage of cells in phase S. Finally, a blockage in phase G2/M occurred in both cell lines and at all treatment times, although it was more evident in SK-N-Be(2) cells (Figure 5).

**Figure 6:**
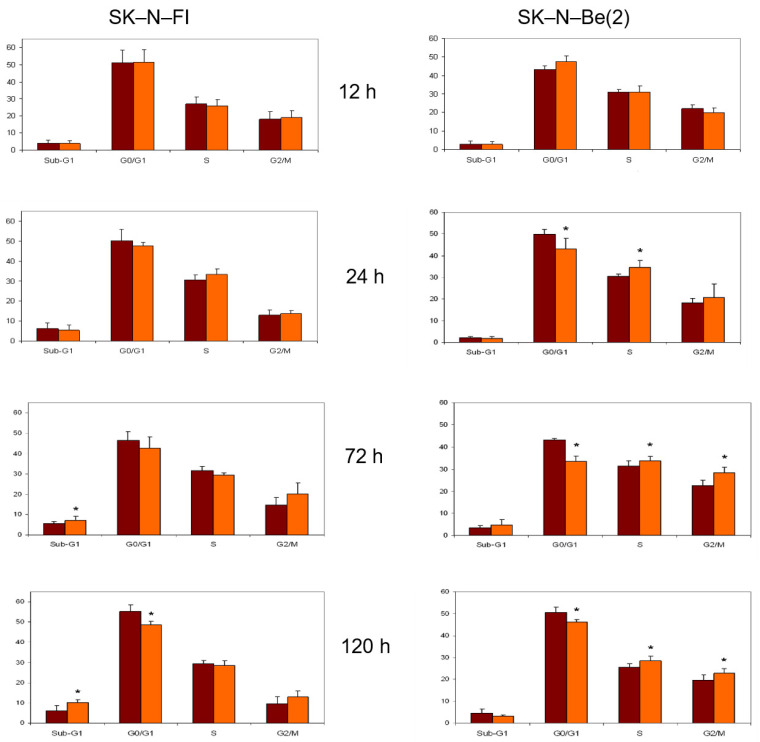
Effect of 1.5 μM cisplatin treatment on cell cycle distribution in SK-N-FI and SK-N-Be(2) neuroblastoma cell lines. Histogram that collects the aggregated results of at least three independent experiments performed in duplicate. Results are expressed as mean ± SEM. * *p* < 0.05. Dark column: control; clear column: cisplatin.

Treatment with cisplatin at a concentration of 1.5 µM caused a decrease in the percentage of cells in G0/G1 phase in both lines. With respect to the S phase, an increase in the percentage of cells was detected in the SK-N-Be(2) cell line but no changes were observed in SK-N-FI cells. Finally, an increase in cells in the G2/M phase was identified after 72 h of treatment in both cell lines (Figure 6).

**Figure 7:**
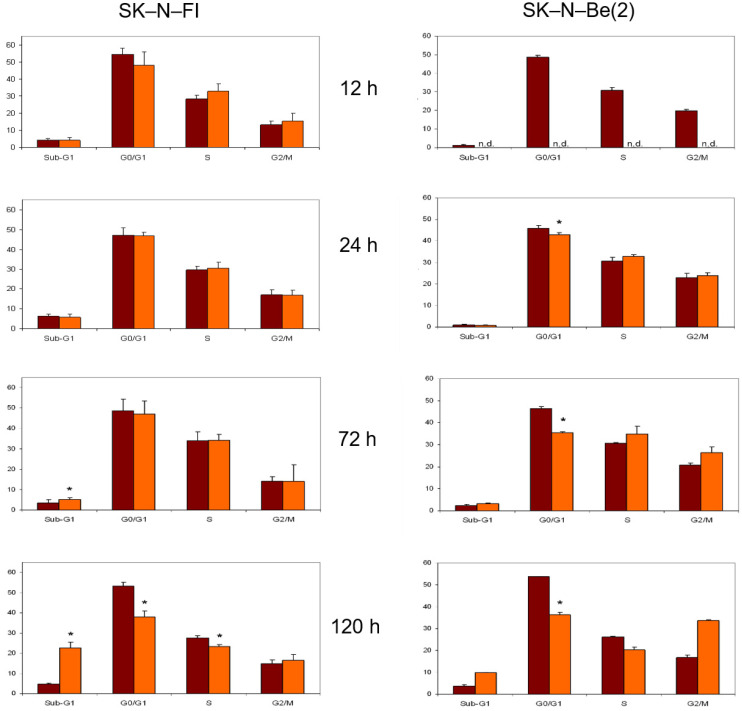
Effect of 0,3 μM melphalan treatment on cell cycle distribution in SK-N-FI and SK-N-Be(2) neuroblastoma cell lines. Histogram that collects the aggregated results of at least three independent experiments performed in duplicate. Results are expressed as mean ± SEM. * *p* < 0.05. Dark column: control; clear column: melphalan.

Regarding the chemotherapeutic agent melphalan, we observed a decrease in the percentage of treated cells in both G0/G1 and S phases at long times, and a blockage of the cell cycle in G2/M only in SK-N-Be(2) cells (Figure 7).

**Figure 8:**
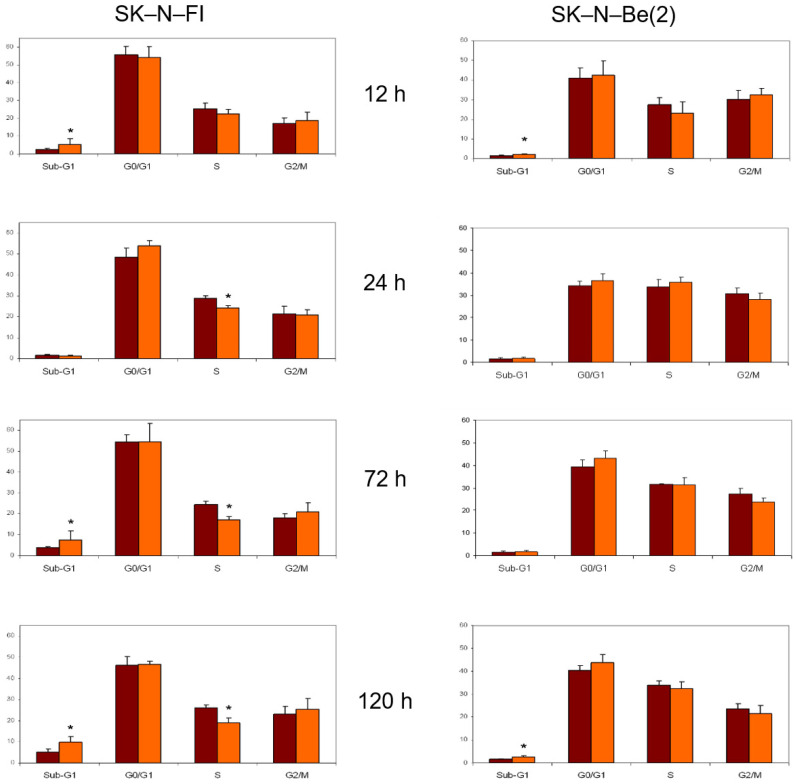
Effect of 1 μM 9–cis-RA treatment on cell cycle distribution in SK-N-FI and SK-N-Be(2) neuroblastoma cell lines. Histogram that collects the aggregated results of at least three independent experiments performed in duplicate. Results are expressed as mean ± SEM. * *p* < 0.05. Dark column: control; clear column: 9–*cis*-RA.

**Figure 9:**
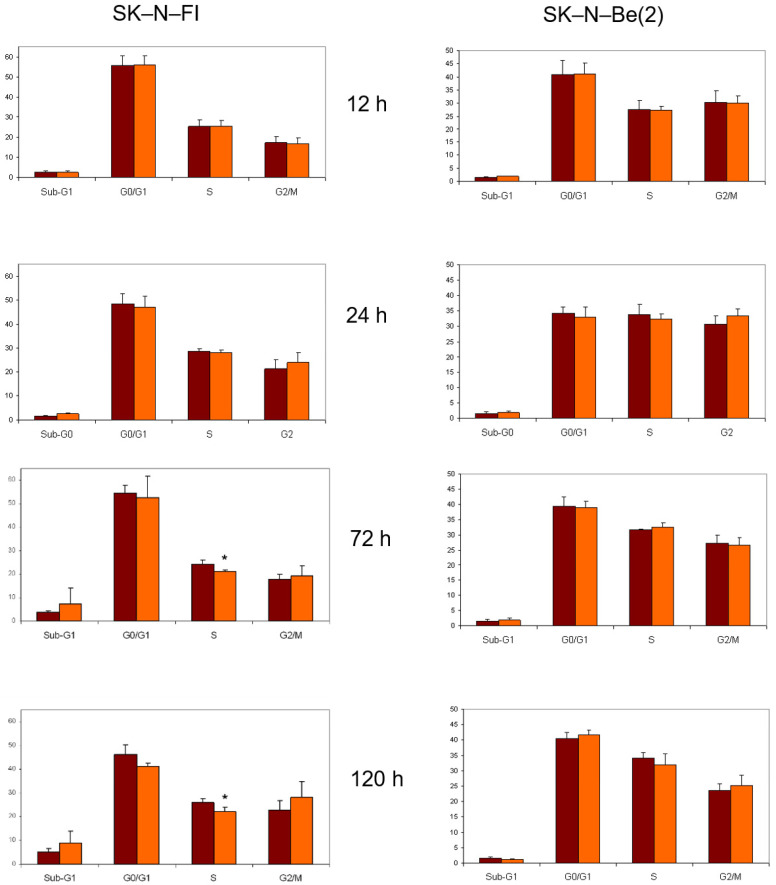
Effect of 1μM *at*-RA treatment on cell cycle distribution in SK-N-FI and SK-N-Be(2) neuroblastoma cell lines. Histogram that collects the aggregated results of at least three independent experiments performed in duplicate. Results are expressed as mean ± SEM. * *p* < 0.05. Dark column: control; clear column: *at*-RA.

Finally, treatment with either of the two isoforms of retinoic acid affected the distribution of the cell cycle only in SK-N-FI cells; a decrease in the percentage of cells in S phase was observed without modifying the percentage of cells in G0/G1 or G2/M phases (Figure 8 and Figure 9).

### 2.9. Protein Expression Analysis

Immunofluorescence detection of Bcl-2, p53, and p21 proteins was carried out in SK-N-FI and SK-N-Be(2) cells after 24 h of treatments with the corresponding compounds.

**Figure 10:**
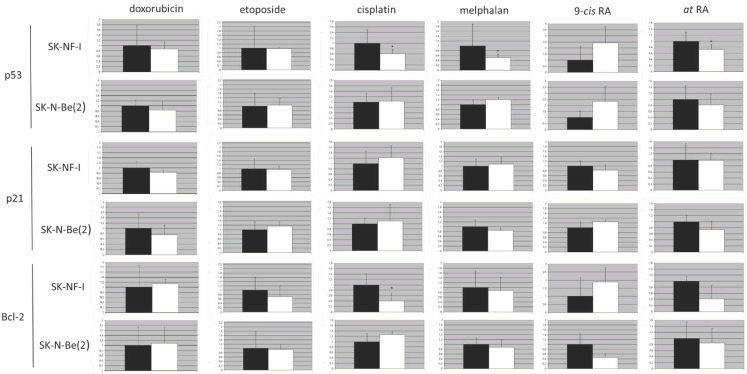
Effect on the relative expression of the p53, p21, and Bcl-2 proteins after treatments, for 24 h. Neuroblastoma cells were treated with doxorubicin, etoposide, cisplatin, melphalan, 9-*cis*-RA, and *at*-RA. Relative expression of p53, p21, and Bcl-2 proteins was determined afterwards. Bar graph indicating the mean values of expression (white color) relative to those of the control cells (black color). To obtain the results, at least four independent experiments were carried out in duplicate. Values are expressed as mean ± SEM. * *p* < 0.05.

Doxorubicin (at 0.2 µM concentration) did not significantly modify the expression of p53, p21, or Bcl-2 in either of the two cell lines analyzed (Figure 10).

Etoposide (0.5 µM) did not modify the expression of p53 and p21 proteins, in either of the two cell lines (Figure 10). However, as a consequence of this treatment, a decrease of around 40% in the levels of Bcl-2 expression was observed in the SK-N-FI cell line. However, probably due to the variability between experiments, statistical significance for this result was not observed.

Treatment with cisplatin (1.5 µM) reduced by about 33% (*p* < 0.05) the expression levels of the p53 protein in SK-N-FI cells (Figure 10). It also reduced Bcl-2 levels in this cell line by around 50% (*p* < 0.05). However, this treatment did not modify the expression of these proteins in SK-N-Be(2). The treatment did not affect the levels of p21 in either of the two cell lines.

The analysis of the expression of p53 in cells exposed to the chemotherapeutic agent melphalan (0.3 µM) revealed a statistically significant decrease (reducing its expression by half, with respect to the control) of the protein in SK-N-FI cells, mutated for TP53. This reduction was not observed in SK-N-Be(2) cells. In the case of p21, practically no alterations were observed, although p21 expression decreased in SK-N-Be(2) cells. Finally, in none of the neuroblastoma cell lines was the expression of Bcl-2 modified significantly as a consequence of the treatment with melphalan (Figure 10).

Retinoic acid, in its two isoforms, was the compound that, when administered in both cell lines, produced the most obvious variations in the expression of the three proteins. Nevertheless, we should take into account that data were not statistically significant, although they showed a clear and repetitive tendency. The 9-*cis*-RA (1 µM) gave rise to a clear increase in the expression of p53 in both SK-N-FI and SK-N-Be(2) cells, presenting levels more than two times higher than the control cells. However, there were opposite variations regarding p21 and Bcl-2. A decrease in p21 expression was detected in the SK-N-FI cell line, the opposite effect to that found in SK-N-Be(2) cells. Again, wide variations were estimated for the Bcl-2 protein, whose expression increased in SK-N-FI almost twice and decreased in SK-N-Be(2) to less than half (Figure 10). Finally, alterations were observed in the case of p53 expression in cells treated with *at*-RA (1 µM): a decrease in p53 protein expression was observed in both cell lines, which was statistically significant in the case of SK-N-FI. Treatment also decreased, although not significantly, the levels of p21 in SK-N-Be(2) cells and those of Bcl-2 in both cell types (Figure 10).

## 3. Discussion

Given that many chemotherapeutic drugs induce DNA damage, there is an interest in determining whether the p53 status in tumors is useful for predicting chemosensitivity [25]. Neuroblastomas usually contain a normal TP53 gene, which is consistent with their chemosensitive phenotype [26]. Mutations of TP53 are rare in neuroblastoma [16], but loss of p53 function results in genomic instability so that tumor cells either fail in their attempt to stop at G1 or reduce their apoptotic capacity, which may play an important role in chemoresistance [27,28,29]. For this reason, in this work we tried to analyze whether drug resistance in neuroblastoma cell lines is associated with a lack of p53 function and/or with TP53 mutations. Topoisomerase inhibitors (etoposide [30] and doxorubicin [31]) and DNA alkylating substances (cisplatin [32] and melphalan [33]) are chemotherapeutic agents currently administered to neuroblastoma patients. We used these drugs in our study, which we have performed with tolerable doses in vivo [4]. These drugs cause toxicity, eventually leading to DNA double strand breakage, resulting in p53 activation, cell cycle arrest, and induction of apoptotic cell death. Together with these drugs, we also analyzed the effect produced by 9-*cis*-RA and *at*-RA, also used clinically [9]. DNA damage induced by different genotoxic agents causes different molecular and cellular responses in established cell lines that have divergent molecular attributes [25]. Out of a total of 10 neuroblastoma cell lines, as a starting point for our study, we chose SK-N-FI and SK-N-Be(2) cell lines, with N and S phenotypes, respectively. Depending on the status of p53, we found clear differences in the response of the two cell lines to the different chemotherapeutic agents. SK-N-FI cells, which had a mutated TP53 gene, were generally more resistant to treatments.

Tweddle et al. observed that the presence of a TP53 mutation in SKNBE(2c) cells is at least one of the chemo and radioresistance mechanisms in this cell line [34]. Furthermore, in vitro studies performed with neuroblastoma cell lines have shown that cells with mutated TP53 and cells with p53 inactivated by overexpression of the viral oncoprotein E6 are less sensitive to chemotherapy with carboplatin, melphalan, and etoposide [28,35] than those expressing functional p53 [13]. Resistance has also been observed, both in vitro and in vivo, to doxorubicin, cisplatin, and vincristine as a result of the inactivation of p53 [13,36]. Despite this, it should be remembered that drug-resistant cell lines with normal and functional p53 have also been described. Therefore, it has been suggested that alterations in cell growth inhibition and p53-dependent apoptosis pathways, other than p53 loss of function, could mediate the level of drug resistance in neuroblastoma [37,38], including an abnormal cytoplasmic accumulation of wild-type p53 [39].

Gain of function in some types of tumors is believed to be a mechanism by which mutated TP53 contributes to tumor progression and increased resistance to chemotherapy and ionizing radiation [36,40,41]. There are currently clinical data to support both types of positive and negative correlations between TP53 mutations and drug susceptibility of tumors [36].

Retinoic-acid-induced differentiation of neuroblastoma cell lines is associated with a reduction in p53 expression [15,42]. Treatment with retinoic acid also reduces MYCN expression [43,44] and increases p27 expression [45]. Ectopic expression of MYCN inhibits retinoic-acid-induced differentiation in neuroblastoma cells [44]. MYCN has been suggested to be a candidate therapeutic target [46,47] since its suppression could provide an alternative to cytotoxic therapy for relapsed neuroblastoma with an inactivated p53 pathway [44].

If p53 alterations occur frequently in relapse, or if chemoresistance is associated with loss of p53 function, the use of chemotherapeutic agents in neuroblastoma that either do not depend on the presence of a functional p53 (taxol, ceramide modulators, BSO/L-PAM) or are more effective if TP53 is inactivated would be highly recommended [15,37]. Our results support this idea since we have observed induction of apoptosis by treatment with 9-*cis*-RA in the SK-N-FI cell line that has an inactivating p53 mutation, but not in the SK-N-Be(2) cell line that expresses normal p53.

The lack of accord between RT-PCR data and Western Blot data about p14ARF expression might be clarified by the following hypotheses [48]. Maugeri et al. [49] demonstrated that several miRNAs participate in neuroblastoma as tumor suppressors (miR-29a-3p, miR-34b-3p, miR-181c-5p, and miR-517a-3p). miRNA appeared to be downregulated in neuroblastoma cell lines due to promoter methylation, but treatments with 5′azacitidine increased miRNA expression and led to malignancy decrease through downregulation of miRNAs’ targets such as CDK6, DNMT3B, E2F3, OLFM3, and IFNAR1.

Methylation of RNA would be another possible explanation for the discrepancies of p14ARF expression data obtained by RT-PCR and Western Blot [50,51,52]. Nachtergaele and He [50] reviewed the molecular mechanisms of translational control. Modifications can occur at any kind of RNA, such as miRNA, lncRNA, mRNA, and others. N-6-methyladenine is the best characterized modification in RNA, demonstrating a role in mRNA decay and translation [51]. The localization of the modification in a transcript, at the 5′UTR or 3′UTR, might make the same protein coordinate with different cellular machinery and result in very different outcomes. Furthermore, writer, eraser, and reader proteins of N-6-methyladenine have been discovered [52], with N-6-methyladenine-dependent mRNA processing promoting translation and decay and affecting splicing.

Circular RNAs might also be considered as regulators of translation [53,54,55,56]. CircRNA-TBC1D4, circRNA-NAALAD2, and circRNA-TGFBR3 may be tumor suppressor genes in neuroblastoma. If downregulated simultaneously, they may serve as biomarkers of unfavorable neuroblastoma [53]. Other circular RNAs might act as oncogenes: circ_0132817 facilitates cell proliferation, migration, invasion, and glycolysis in neuroblastoma [54]; circPDE5A is upregulated in neuroblastoma tissues and cells and its silencing suppresses neuroblastoma cell proliferation, migration, invasion, and glycolysis in vitro and reduces tumor growth in vivo [55]; circular RNAs with oncogenic properties might even result from MYCN amplification in neuroblastoma cells [56].

We analyzed the effect of different antitumor drugs and observed that they have cytotoxic activity at concentrations analogous to those observed in plasma of patients under treatment. Our results show higher apoptosis levels after cisplatin treatment in SK-N-FI cells, despite having mutated TP53, than in SK-N-Be(2) cells.

The loss or partial retention of functions and the gain of new activities probably explain the high heterogeneity of cells that express mutated TP53, also with respect to apoptosis. TP53 mutations have been associated with a poor clinical prognosis and resistance to apoptosis-inducing drugs, but proapoptotic activity has also been shown in some cells with mutated TP53 [57].

Paffhausen et al. [4] suggested that MYCN-expressing cells have a greater sensitivity to drug-induced cell death. In fact, their experiments showed that treatment with increasing drug concentrations leads to apoptosis synergistically along with induced MYCN expression. MYCN expression also correlates with an increase in topoisomerase alpha II expression, which explains the increased sensitivity to topoisomerase II inhibitors such as etoposide and anthracyclines in MYCN-expressing cell lines [58].

In our work we have observed MYCN amplification in the SK-N-FI cell line. This could be one reason that explains the greater apoptotic effect of etoposide in these cells compared to the rest of the chemotherapy drugs analyzed. Keshelava et al. [35] have also described a greater sensitivity to etoposide than to cisplatin and carboplatin in other neuroblastoma cell lines. Modulation of MYCN expression also increases the sensitivity of neuroblastoma cells to other drugs such as vincristine and doxorubicin [58].

Furthermore, caspase-8-deficient neuroblastoma cells are known to be resistant to apoptosis mediated by death receptors and by doxorubicin [59]. However, the resistance to apoptosis manifested by SK-N-FI and SK-N-Be(2) cells, analyzed in this work, is not due to deficiencies in the expression of caspase 8 [60].

The presence of TP53 mutations in cancer cells could not only affect the apoptotic response to treatment but could also allow the cell with genomic damage to enter S phase by preventing cell cycle arrest. In fact, in certain cell types, and in response to some agents that damage DNA, the repair role of p53 could be more important than the induction of apoptosis by p53 [25]. This observation agrees with the results we obtained by flow cytometry analysis of the cell cycle. Our results show a greater capacity of the drugs to affect the cell cycle in SK-N-Be(2) than in SK-N-FI cells, which is probably due to SK-N-Be(2) expression of wild-type p53.

Different neuroblastoma cell types have been reported to have a decreased ability of p53 to block the cycle in G1, although they express normal and functional p53 and do not have affected apoptosis mediated by p53 [26]. It has also been reported that IMR-32 and NGP cell lines, which express wild-type p53, cannot induce G1 stop after irradiation, despite evidence of p21 induction. These cells undergo, surprisingly, a G2 shutdown.

In this work we have also observed a lower capacity of cells, both SK-N-FI and SK-N-Be(2), to stop in the G1 phase after treatment with chemotherapeutic agents, a result that occurs without modifications in the expression of p21. However, after treatment with retinoic acid, a differentiating agent, we did not observe changes in the distribution of the cell cycle in any of the cell lines analyzed, despite the fact that 9-*cis*-RA treatment was accompanied by increases in the expression of p53 that correlated with a greater apoptotic capacity of the cells. Similar results have been described by Xue et al. [61], who have suggested that the response of neuroblastoma cells that express functional p53 after DNA damage depends on the amount of damage and the cell type in question: at high doses of radiation, N-type IMR-32 cells undergo apoptosis rather than cell cycle stop, while S-type SH-EP cells experience senescence.

These authors also observed similar results when treating cells with doxorubicin [36]. Retinoic acid treatments of MYCN-amplified neuroblastoma cells have also been reported to cause differentiation and G1 arrest of these cells, although overexpression of MYCN counteracts G1 stop and retinoic-acid-induced differentiation. For this reason, it has been suggested that retinoic acid causes arrest in G1 through MYCN-dependent and independent mechanisms [44,46].

We have analyzed whether treatments with antitumor agents of neuroblastoma cell lines that have a functional (SK-N-Be(2)) or mutated (SK-N-FI) TP53 gene modify the levels of p53 protein in these cell lines. Our results show that treatments with doxorubicin and etoposide do not significantly modify p53 levels in either of the two cell lines. Cisplatin and melphalan treatments did not modify p53 levels in SK-N-Be(2) cells, but decreased them in SK-N-FI cells. This decrease could be the consequence of the TP53 mutation in this cell line, manifested with a lower capacity of these cells to stop their growth in G1 and a greater capacity to enter apoptosis. Furthermore, the expression levels of p21 in these cells are not modified by the treatment. This result suggests that the mutation does not completely suppress the transactivating capacity of p53, although it attenuates it, as described in other cases [37]. Despite this, it should be remembered that other studies carried out with the compound melphalan have obtained results opposite to those described here [28,37].

Interestingly, retinoic acid treatments led to increases in p53 expression when 9-*cis*-RA was used and to decreases in p53 expression when *at*-RA was used. This result may perhaps explain why 9-*cis*-RA treatment leads to apoptosis, while *at*-RA treatment preferentially causes cell differentiation. In fact, low levels of p53 expression have been detected in differentiating neuroblastomas and it has been reported that retinoic-acid-induced differentiation of neuroblastoma cells reduces p53 levels in them and that pretreatment of neuroblastoma cell lines with retinoic acid confers on these cells resistance to p53-dependent apoptosis [62].

It has been suggested that the most frequent event that confers high-level chemoresistance in neuroblastomas is a loss of function of p53 [28]. In this work we have shown that SK-N-FI cells respond to treatments with various antitumor agents, suggesting that p53 functions are retained, at least in part, despite the mutation detected. Our results suggest that this mutation does not affect the general chemosensitivity of the cells, although it manifests with alterations in the cell cycle. Along with the residual activity of p53, another possible explanation for our results would be that the effects of these compounds were exerted through their homologues p63 and p73 in cells with mutated TP53, also capable of suppressing cell growth and inducing apoptosis. Finally, it is also possible that the effects of the drugs on these cells are due to independent activities of p53 [63]. Chen et al. have reported that MYCN stimulates the transactivating capacity of p53, leading to increased expression of its target genes. Furthermore, these authors have shown that the expression of p53 correlates with that of MYCN in tumor samples and neuroblastoma cell lines [64]. The amplification of MYCN in SK-N-FI is another possible explanation for the effects of p53 in this cell line.

Doxorubicin treatment has been reported to induce p53-mediated expression of p21 in a mouse model [19]. Doxorubicin treatment also induces p21 expression in neuroblastoma cell lines in which the p53/MDM2/p14ARF pathway is not altered, while cell lines with mutated TP53 are not capable of raising p21 levels above their basal levels [61]. We have not been able to observe changes in p21 expression levels after doxorubicin, etoposide, cisplatin, or melphalan treatments of cell lines SK-N-FI or SK-N-Be(2).

However, we detected an inability of cells to stop their growth in the G1/S transition in response to treatments. Dysfunction in the G1 checkpoint of the cell cycle as a consequence of direct mutations of TP53 or of other tumor suppressor genes such as p21 and Rb is widely documented [15,26,28,34,61]. However, a deficiency in the G1/S checkpoint has also been described in neuroblastoma cells with normal p53, although p21 is induced [26,36,62].

p53 can also trigger a stop of the cycle in G2/M, through inhibition of CDK1 [65]. It is therefore of interest that we have detected a blockage of the cell cycle in G2 after treatments of SK-N-FI and SK-N-Be(2) cells with doxorubicin, etoposide, cisplatin, and melphalan, since this result suggests that these cells have a preferential option for this pathway. MYCN amplification could be another explanation for this result since neuroblastoma cells that express normal p53 and have amplified MYCN have been reported to be unable to stop in G1, despite induction of p53, p21, and MDM2 after damage to DNA [62,66]. In addition, MYCN amplification is associated with reduced levels of expression of p21 [44]. Therefore, it has been proposed that MYCN affects the G1 checkpoint through p53-dependent and independent mechanisms: it would decrease the levels of p21 through p53, and it would affect the G1 checkpoint in unstressed cells by independent mechanisms of p53 [67]. Moreover, the response to DNA damage of neuroblastoma cell lines that express wild-type p53 and have MYCN amplified depends on the cell type. Thus, N-type cells would have a lower capacity to block the cell cycle in G1 than S-type cells, since they express lower levels of p21.

We did not detect alterations in the cell cycle after treatment with the different isomers of retinoic acid. A decrease in p21 was observed in SK-N-FI cells after treatments with 9-*cis*-RA and in SK-N-Be(2) cells after treatments with *at*-RA. However, it is noteworthy that 9-*cis*-RA raised p21 levels in SK-N-Be(2) cells without stopping the cell cycle in G1, which suggests that p21 might not be functional in SK-N-Be(2) cells. Although p21 mutations are rare in human tumors, those that have been identified appear to be incapable of inhibiting CDK activity and blocking the cell cycle in G1. Possible alternatives to explain the attenuation of G1 arrest include amplification of cyclin D1, cdk4, cdk6, and pRb mutations. These possible alterations have been described in neuroblastoma, although their frequency is low [26].

Bcl-2 is strongly expressed in most primary tumors and neuroblastoma cell lines, and its expression levels are inversely correlated with the proportion of cells that undergo apoptosis and with the degree of cell differentiation [59]. It has been proposed that p53 represses Bcl-2 transcription. High levels of Bcl-2 expression have also been correlated with tumor severity, which is associated with unfavorable histology, MYCN amplification, and poor prognosis [68].

We have observed a decrease in the levels of Bcl-2 expression that correlates with an increase in apoptosis in the SK-N-FI cell line after 24 h of treatment with cisplatin and/or etoposide. This result supports the idea that p53 functions, despite the mutation of the protein, are conserved, at least partially, in this cell line. It also suggests the correct functioning of the intrinsic pathway of apoptosis in these cells. On the contrary, the reduction in the expression levels of Bcl-2 without induction of apoptosis observed in SK-N-Be(2) cells after treatment with retinoic acid suggests a defect in the intrinsic pathway of apoptosis. The regulation of apoptosis is another possibility to explore in targeted therapies: the analyses of the effect of G3139, an antisense of Bcl-2, and of obatoclax, a pan-inhibitor of the Bcl-2 family, are current examples of these analyses [3].

## 4. Materials and Methods

### 4.1. Cell Lines

A panel of 10 neuroblastoma cell lines established from patients at different stages of therapy and the glioma cell line SW1783 as a control were used. Five of them (SK-N-DZ, SK-N-SH, SK-N-Be(2), SK-N-FI, and Be(2)C) were obtained from the American Type Culture Collection (Manassas, VA, USA), while the remaining five (IMR-32, Kelly, SIMA, SH-SY5Y, and MHH-NB-11) were obtained from the Leibniz Institute DSMZ-German Collection of Microorganisms and Cell Cultures GmbH (Braunschweig, Germany). SK-N-MC (DSMZ, Braunschweig, Germany) and MC-IXC (ATCC, Manassas, VA, USA) cell lines were also studied. SK-N-MC is a neuroepithelioma cell line that today is considered to be derived from an Askin’s tumor, morphologically similar, related to Ewing’s sarcoma [69]. MC-IXC is a cell line subcloned twice from the SK-N-MC cell line which was established in September of 1971 from a metastatic tumor mass (https://www.atcc.org/products/crl-2270#detailed-product-information, accessed on 11 May 2010). These two cell lines were considered to be neuroblastoma cells some years ago. Finally, the glioma cell line SW1783 (ATCC, Manassas, VA, USA) was used as a positive control for the expression of the p14ARF protein.

The 10 neuroblastoma cell lines, SK-N-MC, and MC-IXC were cultured in Dulbecco’s modified Eagle’s medium (DMEM+ L-Glutamax, Invitrogen, Life Technologies, Carlsbad, CA, USA), supplemented with 10% heat-inactivated fetal bovine serum (Invitrogen, Life Technologies, Carlsbad, CA, USA) and 5% non-essential amino acids (Invitrogen, Life Technologies, Carlsbad, CA, USA) in a 37 °C, 5% CO_2_ humidified incubator. In addition, 1% penicillin/streptomycin as antibiotic (Invitrogen, Life Technologies, Carlsbad, CA, USA) and 0.1% amphotericin B as antifungal agent (Sigma-Aldrich Co, Saint Louis, MO, USA) were added. SW1783 glioma cell line was grown in RPMI L-Glutamax (Invitrogen, Life Technologies, Carlsbad, CA, USA), 10% heat-inactivated fetal bovine serum, 1% penicillin/streptomycin (Invitrogen, Life Technologies, Carlsbad, CA, USA), and 0,1% amphotericin B (Sigma-Aldrich Co, Saint Louis, MO, USA).

### 4.2. p14ARF Gene Promoter Methylation

#### 4.2.1. DNA Extraction and Bisulphite Treatment

DNA from the cell lines was purified by the Wizard Genomic DNA Purification Kit (Promega Corporation, Madison, WI, USA) according to manufacturer’s instructions. In addition, 1 μg genomic DNA was bisulphite modified by the CpGenome™ DNA Modification Kit (Chemicon International Inc., Temecula, CA, USA), following the manufacturer’s protocol. Briefly, genomic DNA was denatured by NaOH with a final concentration of 420 mM at 37 °C for 10 min. DNA was modified by adding 550 μL of 3M sodium bisulphite (pH 5.0) and incubating it for 16 h at 50 °C. The bisulphite-treated DNA was washed with 90% ethanol, precipitated, resuspended in 1 mM TE pH 8, and stored at −80 °C until use. Peripheral blood genomic DNA from a healthy donor and in vitro methylated DNA (CpGenome™ Universal Methylated DNA, Chemicon International, Inc., Temecula, CA, USA) were used as negative and positive controls, respectively, for the methylation status of DNA.

#### 4.2.2. Methylation-Specific PCR

For the analysis of the methylation pattern in the CpG islands of the promoter of the P14ARF gene we used methylation-specific PCR (MSP). Bisulphite-modified DNA was amplified by PCR using two primer sets specific for methylated (M) and unmethylated (U) sequences of the p14ARF gene previously described (Table 3). The methylation-specific PCRs were carried out with 120 ng of bisulphite modified DNA in a total volume of 50 μL. PCR mixture also contained 5 μL 10× reaction buffer, 2.5–3 mM MgCl_2_, 0.2 mM of each dNTP, 15 pmol forward and reverse primers, 5% DMSO, and 1 U BioTaq™ DNA polymerase (Bioline Ltd., London, UK). PCR reactions were carried out in a PTC-200 Peltier Thermal Cycler (MJ Research, Watertown, MA). PCR reactions were denatured at 95 °C for 11 min, followed by 35 cycles of 30 s at 94 °C, 30 s at the annealing temperature of 62 °C, 30 s at 72 °C, followed by a final extension step at 72 °C for 10 min. PCR products were visualized in a 2% agarose gel stained with ethidium bromide at a final concentration of 0.1 μg/mL.

#### 4.2.3. Melting Curve Analysis–Methylation Assay

Melting curve analysis–methylation assay (MCA-Meth) was performed with 6 ng of bisulphite modified DNA in a total volume of 25 μL, containing 12.5 μL of Brilliant SYBR Green Master Mix (Stratagene, La Jolla, CA, USA) and 2.5 pmol of primers, in an IQ5 Multicolor Real-Time PCR Detection System (Bio-Rad, Hercules, CA, USA). MCA-Meth primers were designed using the MethPrimer software [70], lacking CpG-dinucleotides to facilitate amplification independence of the methylation status of the amplified target DNA (Table 3). The reactions were heated at 95 °C for 10 min, followed by 45 cycles of 30 s at 95 °C, 30 s at the corresponding annealing temperature, and 30 s at 72 °C. Extra steps of 30 s at 95 °C and 30 s at 70 °C were added. After gene amplification, the melting curve analysis was performed as follows: from 70 °C to 90 °C, the temperature was increased 0.5 °C every 30 s.

### 4.3. p14ARF Expression

#### 4.3.1. RNA Extraction and Reverse Transcription

RNA was extracted from cell cultures using the RNeasy Mini Kit (Qiagen, Venlo, the Netherlands), according to the manufacturer’s instructions. Possible contaminating DNA was removed with the Amplification Grade DNase I (Sigma-Aldrich Co, Saint Louis, MO, USA). Total RNA (1 μg) was transcribed to cDNA by using the Superscript II kit (Invitrogen™, Life Technologies, Carlsbad, CA, USA).

#### 4.3.2. Semiquantitative RT-PCR

A fragment of 298 bp of the transferrin receptor (TFR) gene was previously amplified as a control of RNA integrity. Primers used for TFR and p14ARF expression study are described in Table 3. PCR mixture contained 2.5 μL 10× buffer, 0.2 mM dNTP, 1–1.5 mM MgCl_2_, 5% DMSO, 10 pmol forward and reverse primers for p14ARF gene (5 pmol for TFR), 75 ng of cDNA, and 1 U Amplitaq Gold™ (Applied Biosystems, Foster City, CA, USA) in a final volume of 25 μL. The reaction conditions of 95 °C for 10 min, 43 cycles of 1 min denaturation at 94 °C, 45 s annealing at 62 °C, 45 s extension at 72 °C, and final extension step for 10 min at 72 °C were used to amplify p14ARF. For TFR, the reactions were cycled with a 10 min initial denaturation step at 95 °C, followed by 30 cycles of 45 s denaturation at 94 °C, 45 s annealing at 60 °C, 2 min extension at 72 °C, and 10 min final extension at 72 °C. PCR products were loaded in an ethidium-bromide-stained 2.5% agarose gel and were subjected to electrophoresis.

#### 4.3.3. Protein Extraction

Briefly, neuroblastoma cell line pellets were resuspended in ice-cold RIPA cell lysis buffer (0.1% SDS, 0.1% Triton x-100, 100 mM NaCl, 50 mM Tris-HCl), supplemented with 1 mM EDTA, 1 mM sodium orthovanadate, 10 mM NaF, 0,1% sodium deoxycholate, and 0,1% proteinase inhibitor cocktail (Sigma-Aldrich Co, Saint Louis, MO, USA) at pH 7.2. Proteins were quantified following the BCA Protein Assay-Reducing Agent Compatible kit (Pierce Protein Research Products, Thermo Fisher Scientific Inc., Waltham, MA, USA) by using a spectrophotometer.

#### 4.3.4. Western Blot Analysis

First, 20–30 μg of proteins was separated by SDS-PAGE in 15% polyacrylamide gels, transferred to polyvinylidene difluoride membranes (Hybond P, GE Healthcare, Chicago, IL, USA), and incubated with primary antibody anti-p14ARF (Santa Cruz Biotechnology, Inc., Dallas, TX, USA) and peroxidase conjugate secondary anti mouse antibody IgG (GE Healthcare, Chicago, IL, USA). Immunoblots were then visualized by using enhanced chemiluminescence ECL (GE Healthcare, Chicago, IL, USA). Protein values obtained were normalized regarding the amount of GAPDH.

### 4.4. p14ARF Homozygous Deletions

Differential polymerase chain reaction assays were performed to study homozygous deletions of p14ARF (exon 1-β). A fragment of the GAPDH gene was used as an internal control. The sequences of the primers used are shown in Table 3. PCR was performed in a final volume of 25 μL with 50 ng of DNA, 1.5 mm of MgCl_2_, 0.2 mM dNTPs, 10× buffer, 5 pmol of the corresponding pair of primers for p14ARF and GAPDH, respectively, and 1 U of BioTaq™ DNA polymerase (Bioline Ltd., London, UK). In each PCR, a negative control for homozygous deletions of p14ARF (DNA from blood of healthy donors) was also included to calculate the normal dosage of the target gene. The PCR products were loaded onto ethidium-bromide-stained 15% polyacrylamide gels (19:1) to obtain a higher resolution and therefore be able to differentiate between the bands of each gene.

### 4.5. TP53 Mutations

Genomic DNA extracted from the cell lines was used for sequencing analysis. TP53 exons 5 to 9 were amplified by PCR using primers described by Toguchida et al. [71], whose sequences are listed in Table 4. The reaction mixture contained 2.5 μL 10× buffer, 0.2 mM dNTP, 1.5 mM MgCl_2_, 10 pmol forward and reverse primers, 50 ng DNA, and 1 U BioTaq™ DNA Polymerase (Bioline Ltd., London, UK) in a final volume of 25 μL. Thermal conditions were 30 cycles of 94 °C for 45 s, 65 °C for 45 s, and 72 °C for 45 s, with an initial 10 min denaturation at 95 °C and a final 10 min extension at 72 °C. Control samples were also included in the sequencing of all exons. The PCR products were gel-purified with columns, (Millipore-Sigma, Billerica, MA, USA) and sequenced in both directions at CIMA, University of Navarra.

TP53 sequence results were compared to the reference wild-type sequences from the Genome Browser of the University of California in Santa Cruz (https://genome.ucsc.edu, accessed on 11 May 2010) or deposited in GenBank (accession no. X54156 and U94788) using EMBOSS Pairwise Alignment Algorithms tool.

### 4.6. FISH (Fluorescence In Situ Hybridization) for MYCN and MDM2

#### 4.6.1. Obtaining Cytogenetic Suspensions

To obtain metaphase cytogenetic suspensions of cell lines, mitoses of cells in culture were stopped by adding a volume of 180–260 µL of colchicine solution with a concentration of 10 µg⁄mL (KaryoMAX^®^ Colcemid™ Solution, Invitrogen, Life Technologies, Carlsbad, CA, USA) and incubated in the oven at 37 °C for 2 or 3 h. The medium was then collected in a centrifuge tube, and the trypsin was neutralized once the cells, already washed with PBS, had detached from the flask. After centrifuging and discarding the supernatant with the vacuum pump, a hypotonic shock was caused by slowly adding 5 mL of a 75 mM KCl solution previously heated to 37 °C (12 min, 37 °C). After centrifuging, the supernatant was removed, except for 1 mL, which was homogenized in the vortex and to which 5 mL of 4:1 Carnoy’s fixative (methanol/acetic acid) was added dropwise. The tubes were kept for 10 min on ice before re-centrifuging and removing the supernatant. The fixation process was repeated three times with methanol/acetic acid (3:1), before resuspending the cells and making the extensions on dry, clean slides previously cooled in a refrigerator. The preparations were stored at −20 °C.

#### 4.6.2. Description of the FISH Probes Used

Two centromeric probes, α2 and α12, specific for chromosomes 2 and 12, respectively, and two other probes specific for MYCN on chromosome 2 (2p24.1) and MDM2 on chromosome 12 (12q14.3-q15) were chosen. The centromeric probes were labelled with Spectrum Green fluorochrome and those of MYCN and MDM2 with Spectrum Red. Hybridizations were performed with a pair of centromere/gene probes simultaneously, so that in each of the cell lines a double fluorescent labelling could be produced: the centromeres in green and the MYCN and MDM2 genes in red.

#### 4.6.3. Locus-Specific FISH Probe Design

For the localization of genomic clones that covered the regions of interest, the Genome Browser of the University of California in Santa Cruz (https://genome.ucsc.edu, accessed 11 May 2010) allowed the selection of different BACs that comprised 100–200 Kb fragments of DNA, large enough to be used as probes in FISH studies.

For the study of the MYCN gene, the BACs RP11-744F11, RP11-120J4, and RP11-635A14 were selected, while for the BACs that overlapped the *MDM2* region, the following three were chosen: RP11–77H17, RP11–878N15, and RP11–1152I7. We acquired these artificial chromosomes at Children’s Hospital Oakland Research Institute (http://www.chori.org, accessed on 11 May 2010).

#### 4.6.4. Culture of the FISH Probes

Bacterial clones in *E.coli* (BACs) are supplied on unstable LB agar which limits their viability. A culture was made in solid LB medium (Luria Bertani, Bacto-tryptone 10 g/L, Bacto Yeast Extract 7.5 g/L, and NaCl 10 g/L at pH 7.5) supplemented with chloramphenicol 12.5 µg/mL by loop depletion seeding, at 37 °C overnight. To obtain the DNA, one of the isolated colonies was selected and amplified by growing it in 10 mL of liquid LB supplemented with chloramphenicol, overnight, at 37 °C while shaking at 250 rpm. The plasmids corresponding to the α2 and α12 centromeric probes were amplified by growing them in 12 mL of LB medium supplemented with ampicillin at a concentration of 25 µg/µL at 37 °C, under constant shaking (250 rpm), overnight. For the conservation of the BAC or plasmid clones, they were frozen at −80 °C in LB-glycerol (800 µL of liquid culture and 200 µL of sterile glycerol). Successive cultures were made from these reservoirs. After plasmid miniprep and determination of the DNA concentration and quality, the labelling of the probes and their subsequent hybridization with the cytogenetic suspensions of the cell lines were subcontracted to the company Genetadi Biotech SL (Derio, Vizcaya, Spain), who counted the signals of 100–200 nuclei from each cell line on a Leica DM6000 microscope.

In neuroblastoma, the published guidelines for *MYCN* FISH establish that gene amplification is defined as an increase in MYCN signal greater than four times with respect to the number of chromosomes 2. In turn, gene gain refers to an excess of two to four times in the copy number of MYCN with respect to chromosome 2 [72]. According to this criterion, the SK-N-MC and MC-IXC cell lines showed a normal hybridization pattern for MYCN (Figure S2 [1,2]): colocalization of two green signals (α2) and two red signals (RP11–744F11 BAC) on the same chromosome in most of the nuclei. In the SH-SY5Y and SK-N-SH cell lines, the clone detected mostly showed a 2G 3R hybridization pattern (two green and three red signals), compatible with an MYCN duplication (Figure S2 [3,4]): colocalization of two green and two red signals on the same chromosome and an extra red MYCN signal on an unknown derived chromosome appears in metaphase FISH.

The count of the predominant clones in the BE(2)C, IMR-32 and SK-N-Be(2) cell lines showed two signals of α2 and multiple signals corresponding to RP11-744F11 (2G ampR) which indicates amplification of MYCN on these lines (Figure S2 [5,6,7]). Counting the major clone of SK-N-FI and Kelly cells revealed the existence of nuclei that presented a single green signal, corresponding to the centromere of chromosome 2, and amplification of the red signals of RP11–744F11, which hybridizes with the MYCN gene (1V ampR), suggesting aneuploidy with loss of chromosome 2 (Figure S2 [8,9]). Finally, cell lines with polyploid karyotype (SK-N-DZ, SIMA, and MHH-NB-11) showed MYCN amplification (Figure S2 [10,11,12]): SK-N-DZ and SIMA presented four green signals corresponding to the centromere of chromosome 2 (4V ampR), while in those of MHH-NB-11 the count was 5V ampR.

### 4.7. Detection of Apoptosis

Cell viability of the neuroblastoma lines SK-N-FI and SK-N-Be(2) was quantified by the MTT colorimetric assay (3-(4,5-Dimethyl-2-thiazolyl)-2,5-diphenyl-2*H*-tetrazolium bromide) developed by Mosmann in 1983 [73]. Apoptosis determination and cell cycle analysis were carried out by flow cytometry using the APO-DIRECT™ kit (BD Biosciences, Pharmingen Inc., San Diego, CA, USA) based on the TUNEL method.

The ability of the different drugs to induce apoptosis in SK-N-FI and SK-N-Be(2) cells was determined after 12, 24, or 72 h or 5 days of treatment with doxorubicin (0.2 µM), etoposide (0.5 µM), cisplatin (1.5 µM), melphalan (0.3 µM), 9-*cis*-RA (1 µM), or *at*-RA (1 µM). After the treatments, the cells were trypsinized, collecting the apoptotic bodies from the supernatant by centrifugation. According to the manufacturer’s instructions, they were resuspended for 1 h at 4 °C in 1% (*w/v*) paraformaldehyde (Sigma-Aldrich, Saint Louis, MO, USA) in PBS pH 7.4 (D-PBS, Gibco, Invitrogen, Life Technologies, Carlsbad, CA, USA) at a concentration of 1 × 10^6^ cells/mL. After centrifuging for 5 min at 300× *g* and removing the supernatant, the cells were washed twice with 5 mL of PBS and the pellet was resuspended in the residual PBS by vortexing the tube. Finally, the cells were fixed in ice-cold 70% ethanol adjusting the concentration to 1 × 10^6^ cells/mL, incubated for at least 30 min at 4 °C, and stored at −20 °C.

For labelling, residual ethanol was removed from the supernatant after centrifuging the fixed cells (1 × 10^6^ cells/mL) for 5 min at 300× *g*. After washing the cells twice, they were incubated at 37 °C for 1 h with the labelling solution, which contains the TdT enzyme, FITC-dUTP, and reaction buffer. Rinse solution was added and centrifuged again for 5 min at 300× *g*. This process was repeated by removing the supernatant by aspiration. Finally, the precipitate was resuspended in 0.3–0.5 mL of propidium iodide/RNase solution and incubated for 30 min in darkness at room temperature.

FITC fluorescence was detected at a wavelength of 520 nm, which allowed the apoptosis level of 20,000 cells per sample to be analyzed on the EPICS-XL-4CLR flow cytometer (Beckman-Coulter, Miami, FL, USA), equipped with an argon laser (488 nm). The data, on a logarithmic scale, were analyzed with the EXPO32 ADC software (Beckman Coulter, Miami, FL, USA). Cytometer calibration was checked in each experiment using Flow-Check™ Fluorospheres (Beckman Coulter).

### 4.8. Cell Cycle Analysis

Propidium iodide marks the total DNA of the cells and emits fluorescence at 623 nm, providing additional information on the DNA content and therefore on the phase of the cell cycle in which each cell is. The histogram recording the fluorescence of propidium iodide for DNA content analysis (linear scale) was visualized with the EXPO32 ADC software on 20,000 cells.

### 4.9. Protein Expression

Rabbit polyclonal primary antibodies directed against the N-terminal ends of the Bcl-2 (N-19) and p21 (N-20) proteins and a goat polyclonal primary antibody to detect the C-terminal end of p53 (C-19) (Santa Cruz Biotechnology, Dallas, TX, USA) were used. Isotype antibodies of the different phosphorylated proteins (Santa Cruz Biotechnology, Dallas, TX, USA) were used as negative controls. Subsequently, the experiment was continued by incubation with secondary antibodies bound to FITC, of goat in the case of Bcl-2 and p21, and of donkey in that of p53 (Santa Cruz Biotechnology, Dallas, TX, USA). The samples, in the dark and at 4 °C, were analyzed in the cytometer with no more than 4 h having elapsed since the labeling. Expression analysis was determined from 10,000 cells per sample. The mean fluorescence obtained was subtracted from that corresponding to its negative control to eliminate the nonspecific labeling. At least three independent experiments were carried out in duplicate.

### 4.10. Statistical Analysis

The distribution of samples in apoptosis, cell cycle, and protein expression studies between treated and control cells was analyzed using the Shapiro–Wilks and Kolmogorov–Smirnov tests for normality. Analyses were carried out with the statistical program SPSS 16.0 for Windows (SPSS Inc., Chicago, IL, USA). In the groups in which these tests were significant, a Mann–Whitney U test was performed for non-parametric samples, while in the groups whose distribution was normal, the Student’s t test was performed for parametric samples. The significance limit of the tests was set at 0.05.

## 5. Conclusions

In summary, we have characterized 10 neuroblastoma cell lines for alterations at the p53/MDM2/p14ARF signaling pathway, finding that no cell line presented MDM2 amplification, p14ARF promoter methylation, p14ARF homozygous deletions, or p14ARF expression, while only one of them had a TP53 point mutation. We have also treated two of those cell lines with chemotherapeutic agents (doxorubicin, etoposide, cisplatin, and melphalan) and with two isomers of retinoic acid. SK-N-FI (mutated at TP53) and SK-N-Be(2) (wild-type TP53) cells responded differently to the retinoid isomers. The mutation at TP53 might confer a better response capacity to apoptosis and to stop cell cycle progression, all of which makes us rethink the necessity of deciphering the molecular status of TP53 prior to treating patients of neuroblastoma in order to standardize therapies with the aim of improving survival. As seen here, TP53 does not follow a unique pattern of behavior in cancer cells. Strategies to restore normal function in TP53 mutated tumors, like those of APR-246 (PRIMA-1met) [74,75,76,77,78,79] have been increasing for several years, with promising results in some cases. However, neuroblastoma seems not to be on that line of response: on the contrary, TP53 mutated alleles seem to increase apoptosis and stop the cell cycle without a prior need for wild-type p53 protein restoration. Not only patients with wild-type TP53 neuroblastoma, but also those with mutant TP53 neuroblastoma might have a good outlook if treated with inhibitors of MDM2 [80,81]. In any case, new strategies are needed to solve this issue, with an open look at the p53/MDM2 interaction, in order to clearly improve the prognosis in neuroblastoma patients.

## Figures and Tables

**Table 1:** Summary of the results obtained by FISH analysis of MYCN and MDM2 copy number changes in the corresponding cell lines.

	Hybridization Pattern	
	MYCN	MDM2
SH-SY5Y	2G3R	normal
SK-N-SH	2G3R	normal
BE(2)C	2GampR	3G3R
IMR-32	2GampR	normal
SK-N-Be(2)	2GampR	normal
SK-N-FI	1GampR	normal
Kelly	1GampR	normal
SK-N-DZ	4GampR	5G4R
SIMA	4GampR	4G4R
MHH-NB-11	5GampR	4G4R
SK-N-MC	normal	normal
MC-IXC	normal	3G2R

G: Green, R: Red, ampR: amplification of red signals.

**Table 2:** Genetic profile of the cell lines studied. The table includes information on MYCN and MDM2 amplification, p14ARF methylation, p14ARF expression, p14ARF homozygous deletions, and TP53 point mutations in the cell lines. amp: amplification, meth: methylation, exp: expression, hd: homozygous deletions, mut: point mutations at exons 5–9 of the TP53 gene.

	MYCN amp	MDM2 amp	p14ARF meth	p14ARF exp	p14ARF hd	TP53 mut
SH-SY5Y	−	−	−	−	−	−
SK-N-SH	−	−	−	−	−	−
BE(2)C	+	−	−	−	−	−
IMR-32	+	−	−	−	−	−
SK-N-Be(2)	+	−	−	−	−	−
SK-N-FI	+	−	−	−	−	+
Kelly	+	−	−	−	−	−
SK-N-DZ	+	−	−	−	−	−
SIMA	+	−	−	−	−	−
MHH-NB-11	+	−	−	−	−	−
SK-N-MC	−	−	−	−	−	−
MC-IXC	−	−	−	−	−	−

**Table 3:** Oligonucleotide sequences used for the p14ARF study. F: forward, R: reverse.

Study		Sequence (5′–3′)		Size (bp)
Methylation	MSP	P14ARF U–F:	5′-TTTTTGGTGTTAAAGGGTGGTGTATAGT-3′	132
		P14ARF U–R:	5′-CACAAAAACCCTCACTCACAACAA-3′	
		P14ARF M–F:	5′-GTGTTAAAGGGCGGCGTAGC-3′	122
		P14ARF M–R:	5′-AAAACCCTCACTCGCGACGA-3′	
	MCA–Meth	P14ARF–MET–F:	5′-AAAAATGGGTTAGATATAAAG-3′	248
		P14ARF–MET–R:	5′-CCTCTTCTAAATTTAAAAAACA-3′	
Expression	RT–PCR	P14ARF–RT–F:	5′-CCGCCGCGAGTGAGGGTTTT-3′	242
		P14ARF–RT–R:	5′-GCACGGGTCGGGTGAGAGTGG-3′	
		TFRC–F:	5′-GTCAATGTCCCAAACGTCACCAGA-3′	298
		TFRC–R:	5′-ATTTCGGGAATGCTGAGAAAACAGACAGA-3′	
Homozygous deletion	Differential PCR	P14ARF–DH–F:	5′-TCCCAGTCTGCAGTTAAGGG-3′	174
		P14ARF–DH–R:	5′-ACCACGAAAACCCTCACTCG-3′	
		GAPDH–F:	5′-AACGTGTCAGTGGTGGACCTG-3′	160
		GAPDH–R:	5′-AGTGGGTGTCGCTGTTGAAGT-3′	

**Table 4:** Oligonucleotides used for the analysis of TP53.

Exon	Sequence		Size (bp)
Exon 5 (5′ end)	F:	5′-TTATCTGTTCACTTGTGCCC-3′	189
	R:	5′-TCATGTGCTGTGACTGCTTG-3′	
Exon 5 (3′ end)	F:	5′-TTCCACACCCCCGCCCGGCA-3′	162
	R:	5′-ACCCTGGGCAACCAGCCCTG-3′	
Exon 6	F:	5′-ACGACAGGGCTGGTTGCCCA-3′	201
	R:	5′-CTCCCAGAGACCCCAGTTGC-3′	
Exon 7	F:	5′-GGCCTCATCTTGGGCCTGTG-3′	171
	R:	5′-CAGTGTGCAGGGTGGCAAGT-3′	
Exon 8	F:	5′-CTGCCTCTTGCTTCTCTTTT-3′	204
	R:	5′-TCTCCTCCACCGCTTCTTGT-3′	
Exon 9	F:	5′-GCAGTTATGCCTCAGATTCA-3′	185
	R:	5′-GGCATTTTGAGTGTTAGACT-3′	

F: forward, R: reverse.

## Data Availability

The data presented in this study are available in article.

## Supplementary Materials

The following are available online at https://www.mdpi.com/article/10.3390/ph14111184/s1, Figure S1: p14ARF MCA–Meth results.SK–N–MC (**A**), SK–N–SH (**B**), SK–N–DZ (**C**), Be(2)C (**D**), SK–N–Be(2) (**E**), SK–N–FI (**F**), Kelly (**G**), MHH–NB–11 (**H**), IMR–32 (**I**), SH–SY5Y (**J**) and MC-IXC (**K**). Denaturation curves of the PCR products are shown. Red: DNA extracted from blood cells (unmethylated control). Green: in vitro methylated DNA (methylation control). Blue: the corresponding cell line (*n* = 3). Figure S2-1: MYCN FISH in SK-N-MC cells. The normal MYCN hybridization pattern is shown for this cell line in interphase (**A**) and metaphase (**B**). Green: centromere of chromosome 2; Red: MYCN. Figure S2-2: MYCN FISH in MC-IXC cells. The analysis revealed a normal hybridization pattern, both in interphase (**A**) and metaphase (**B**). Green: centromere of chromosome 2; Red: MYCN. Figure S2-3: MYCN FISH in SH-SY5Y cells. Cells with 2 chromosomes 2 and extra copy (gain) of MYCN is observed in interphase (**A**) and metaphase (**B**) preparations. Green: centromere of chromosome 2; Red: MYCN. Figure S2-4: MYCN FISH in SK-N-SH cells. Most cell line nuclei show 2 α2 signals in green and 3 RP11-744F11 red signals (containing the MYCN gene) in interphase (**A**) and metaphase (**B**). Green: centromere of chromosome 2; Red: MYCN. Figure S2-5: MYCN FISH in BE(2)C cells. Cells with 2 chromosomes 2 and MYCN amplification appear in interphase (**A**) and in metaphase (**B**). Specifically, in metaphase FISH, 2 chromosomes with centromere 2 are observed, in which the MYCN signals are barely seen, masked by the intensity of the amplifications. On the other hand, there are 3 other derived chromosomes that contain an amplification of MYCN in the form of homogeneously stained regions, but without a centromere of 2. Green: centromere of chromosome 2; Red: MYCN. Figure S2-6: MYCN FISH in IMR-32 cells. FISH analysis was carried out on cell extensions in interphase (**A**) and metaphase (**B**). In the metaphase extensions, 2 chromosomes with centromere 2 and the respective MYCN signals are clearly distinguished. Therefore, there is colocalization of the signals in these chromosomes (chromosomes 2 “normal”). In addition, MYCN probe analysis revealed a characteristic homogeneously stained regions shaped gene amplification signal distribution on 2 other derived chromosomes of unknown origin, without chromosome 2 centromere. Green: chromosome 2 centromere; Red: MYCN. Figure S2-7: MYCN FISH in SK-N-Be(2) cells. FISH analysis was carried out on cell extensions at interphase (**A**) and metaphase (**B**). Two chromosomes with centromere 2 and the respective MYCN signals were detected in metaphases, despite not being able to stand out well in the photographs. They are therefore “normal” chromosomes 2. Two other derived chromosomes of unknown origin were also observed showing MYCN amplification in homogeneously stained regions but without the centromere of chromosome 2. Polyploid metaphases were seen in the preparations. Green: centromere of chromosome 2; Red: MYCN. Figure S2-8: MYCN FISH in SK-N-FI cells. Cell nuclei show a pattern of an α2 signal in green and MYCN amplification in red, either in interphase (**A**) or metaphase (**B**) extensions. Green: centromere of chromosome 2; Red: MYCN. Figure S2-9: MYCN FISH in Kelly cells. The only clone of this cell line shows a hybridization pattern of a green signal of the α2 probe (chromosome 2 centromere) and amplification of the signal of RP11-744F11 (containing the MYCN gene), marked in red, in interphase cell extensions (**A**) and in metaphases (**B**). Green: centromere of chromosome 2; Red: MYCN. Figure S2-10: MYCN FISH in SK-N-DZ cells. These cells presented MYCN amplification in interphase (**A**) and metaphase (**B**) preparations. In metaphase FISH, 4 chromosomes with centromere 2 were observed: two of them contained MYCN amplification in homogeneously stained regions, another one showed loss of MYCN, and the last one presented a normal hybridization pattern. In addition, there is another chromosome without a centromere of chromosome 2 and with MYCN amplification in the form of a homogeneously stained region. Green: centromere of chromosome 2; Red: MYCN. Figure S2-11: MYCN FISH in SIMA cells. FISH analysis was carried out on cell extensions in interphase (**A**) and metaphase (**B**,**C**). The analysis carried out indicated the existence of cells with 4 chromosomes 2 and a very large amplification of MYCN (>20 copies) or with a lower degree of amplification of the gene (<20 copies), a difference that can be clearly seen in the photograph of the nuclei in interphase (**A**), showing a diffuse amplification pattern for MYCN. In the metaphase extensions (**B** and **C**), MYCN red signals were observed both inside and outside the chromosomes, compatible with double minute chromosomes. Green: centromere of chromosome 2; Red: MYCN. Figure S2-12: MYCN FISH in MHH-NB-11 cells. FISH analysis was carried out on cell extensions at interphase (**A**) and metaphase (**B**). Multiple copies of MYCN were detected in the interphase extensions (**A**). In metaphase (**B**), colocalization of green and red signals are found on 5 chromosomes and a chromosome of unknown origin that presented a homogeneously stained region amplified MYCN. Green: centromere of chromosome 2; Red: MYCN. Figure S2-13: MDM2 FISH in SK-N-MC cells. Normal hybridization pattern in interphase (**A**) and metaphase (**B**) cell extensions is shown. Green: centromere of chromosome 12; Red: MDM2. Figure S2-14: MDM2 FISH in SK-N-FI cells. Normal hybridization pattern obtained in interphase (**A**) and metaphase (**B**) cell extensions is shown. The metaphase image indicates colocalization of green and red signals on the same chromosome. Green: centromere of chromosome 12; Red: MDM2. Figure S2-15: MDM2 FISH in IMR-32 cells. Normal hybridization pattern found in these cells in interphase (**A**) and in metaphase (**B**) is shown. Green: centromere of chromosome 12; Red: MDM2. Figure S2-16: MDM2 FISH in Kelly cells. All nuclei of the cells analyzed gave a 2Green 2Red count in both interphace (**A**) and metaphase (**B**). Green: centromere of chromosome 12; Red: MDM2. Figure S2-17: MDM2 FISH in SK-N-Be(2) cells. The major clone in these cells presents two signals corresponding to the centromere of chromosome 12 and 2 signals from RP11-77H17 (which contains the MDM2 gene) in interphase (**A**) and in metaphase (**B**). Green: centromere of chromosome 12; Red: MDM2. Figure S2-18: MDM2 FISH in SH-SY5Y cells. All nuclei analyzed revealed, both in interphase (**A**) and in metaphase (**B**), a normal hybridization pattern. Green: centromere of chromosome 12; Red: MDM2. Figure S2-19: MDM2 FISH in SK-N-SH cells. All nuclei analyzed revealed, both in interphase (**A**) and in metaphase (**B**), colocalization of signals corresponding to the centromere of chromosome 12 and to the MDM2 gene. Green: centromere of chromosome 12; Red: MDM2. Figure S2-20: MDM2 FISH in MC-IXC cells. Loss of MDM2 in both interphase (**A**) and metaphase (**B**) cells. Green: centromere of chromosome 12; Red: MDM2. Figure S2-21: MDM2 FISH in BE(2)C cells. Most nuclei present 3 green signals from the centromere of chromosome 12 and 3 red signals corresponding to MDM2 (3G 3R) in interphase (**A**) and in metaphase (**B**). Green: centromere of chromosome 12; Red: MDM2. Figure S2-22: MDM2 FISH in SIMA cells. Counting of the major clone of the cells, in interphase (**A**) and in metaphase (**B**), presented 4 signals of α12 and 4 signals of RP11-77H17 (4G 4R). Green: centromere of chromosome 12; Red: MDM2. Figure S2-23: MDM2 FISH in MHH-NB-11 cells. Images obtained from cell extensions in interphase (**A**) and in metaphase (**B**) are shown. The test showed a 4G 4R result, that is, 4 signals were detected from the centromere of the studied chromosome and another 4 signals from the MDM2 gene, located therein. Green: centromere of chromosome 12; Red: MDM2. Figure S2-24: MDM2 FISH in SK-N-DZ cells. In the interphase nuclei (**A**), 4 centromeres of chromosome 12, and a smaller one and 4 signals of the BAC that hybridizes with MDM2 were observed (5G 4R). Metaphase analysis (**B**) confirmed that these α12 signals were cohybridized with MDM2 on chromosome 12, except for one, which did not show MDM2 hybridization in most nuclei. Green: centromere of chromosome 12; Red: MDM2. Figure S3: Analysis of cytotoxicity induced by doxorubicin, etoposide, cisplatin, melphalan, 9-*cis*-RA and *at*-RA in neuroblastoma cell lines SK–N–FI and SK–N–Be(2). The figure shows the dose-response curves obtained after 72 h of treatment. Data are presented as the mean ± SEM of at least 3 independent experiments. Table S1-1: Status of the SK-NF-I cell line according to the IARC TP53 database (https://p53.iarc.fr/CellLines.aspx; accessed on 8 July 2021). Table S1-2: Neuroblastoma cell lines with a mutated codon 135, as identified in the database.
