# Supplementary material for: In Vitro Assessment of the Role of p53 on Chemotherapy Treatments in Neuroblastoma Cell Lines
Source: Pharmaceuticals (Basel). 2021 Nov 19;14(11):1184. doi: 10.3390/ph14111184 (PMC8624165; doi:10.3390/ph14111184)
Supplement: Supplementary file 1 [file pharmaceuticals-14-01184-s001.zip › Figure S1.pdf]

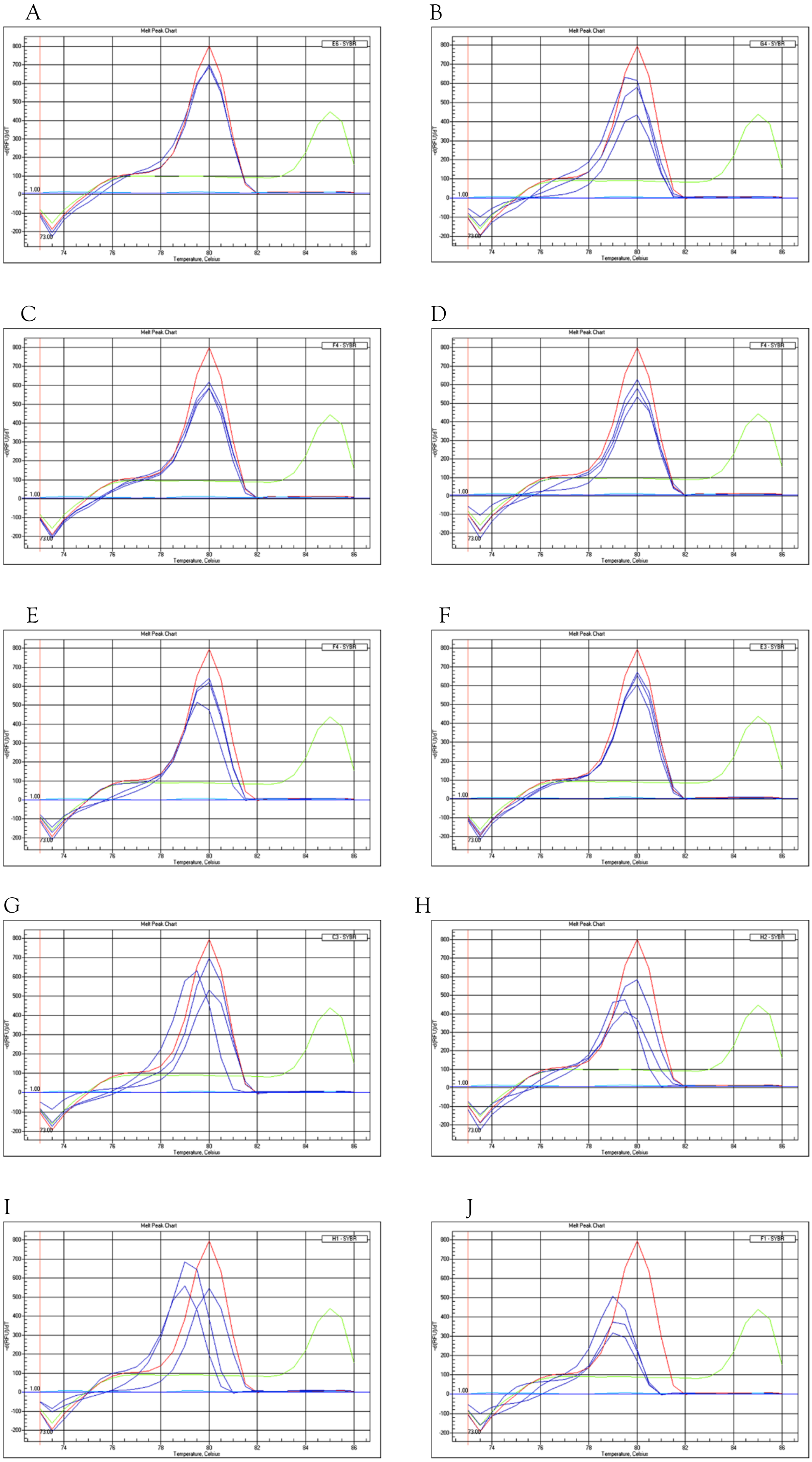
A
Melt Peak Chart
E6 - SYBR
B
Melt Peak Chart
G4 - SYBR
C
Melt Peak Chart
F4 - SYBR
D
Melt Peak Chart
F4 - SYBR
E
Melt Peak Chart
F4 - SYBR
F
Melt Peak Chart
E3 - SYBR
G
Melt Peak Chart
C3 - SYBR
H
Melt Peak Chart
H2 - SYBR
I
Melt Peak Chart
H1 - SYBR
J
Melt Peak Chart
F1 - SYBR
-d(RFU)/dT
Temperature, Celsius

K

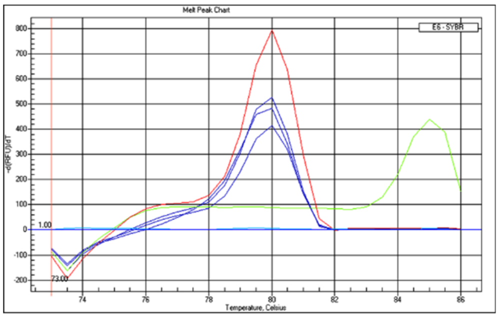

Figure S1. p14ARF MCA–Meth results.SK–N–MC (A), SK–N–SH (B), SK–N–DZ (C), Be(2)C (D), SK–N–Be(2) (E), SK–N–FI (F), Kelly (G), MHH–NB–11 (H), IMR–32 (I), SH–SY5Y (J) and MC-IXC (K). Denaturation curves of the PCR products are shown. Red: DNA extracted from blood cells (unmethylated control). Green: *in vitro* methylated DNA (methylation control). Blue: the corresponding cell line (n = 3).
