# Supplementary material for: In Vitro Assessment of the Role of p53 on Chemotherapy Treatments in Neuroblastoma Cell Lines
Source: Pharmaceuticals (Basel). 2021 Nov 19;14(11):1184. doi: 10.3390/ph14111184 (PMC8624165; doi:10.3390/ph14111184)
Supplement: Supplementary file 1 [file pharmaceuticals-14-01184-s001.zip › Figure S2.pdf]

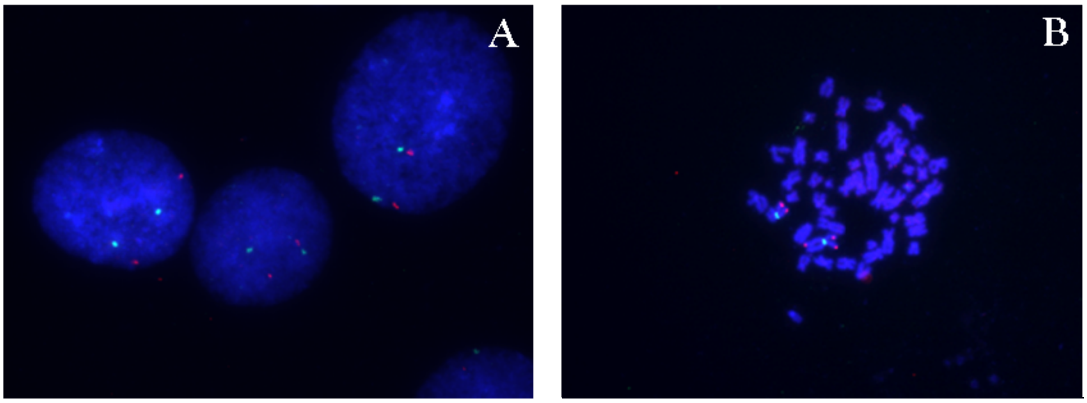

Figure S2-1. ***MYCN* FISH in SK-N-MC cells.** The normal MYCN hybridization pattern is shown for this cell line in interphase (A) and metaphase (B). Green: centromere of chromosome 2; Red: *MYCN*.

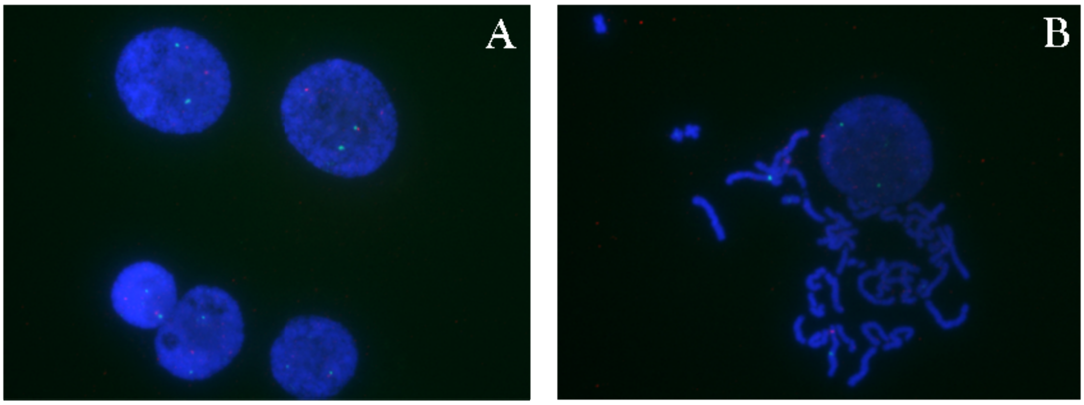

Figure S2-2. *MYCN* FISH in MC-IXC cells. The analysis revealed a normal hybridization pattern, both in interphase (A) and metaphase (B). Green: centromere of chromosome 2; Red: *MYCN*.

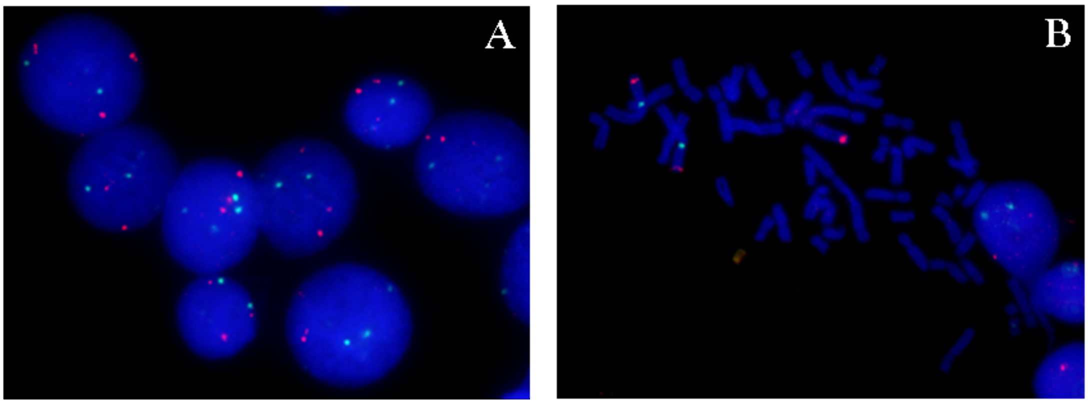

Figure S2-3. *MYCN* FISH in SH-SY5Y cells. Cells with 2 chromosomes 2 and extra copy (gain) of *MYCN* is observed in interphase (A) and metaphase (B) preparations. Green: centromere of chromosome 2; Red: *MYCN*.

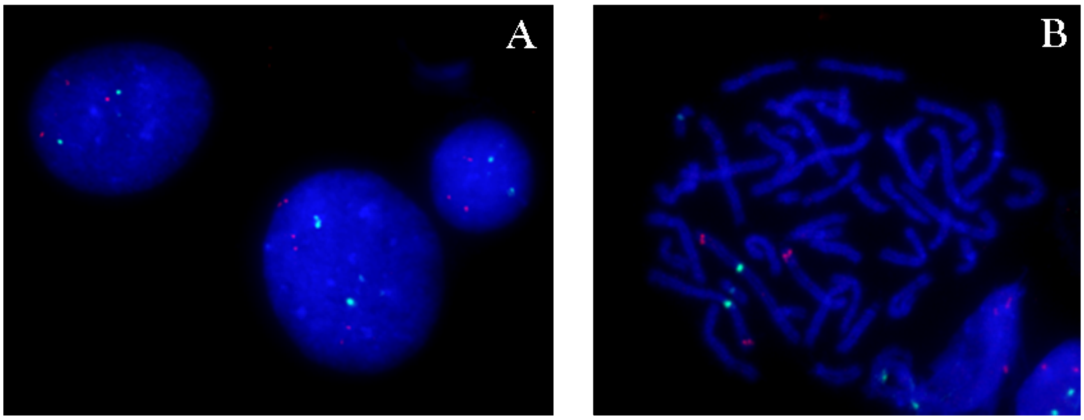

Figure S2-4. *MYCN* FISH in SK-N-SH cells. Most cell line nuclei show 2 α2 signals in green and 3 RP11-744F11 red signals (containing the *MYCN* gene) in interphase (A) and metaphase (B). Green: centromere of chromosome 2; Red: *MYCN*.

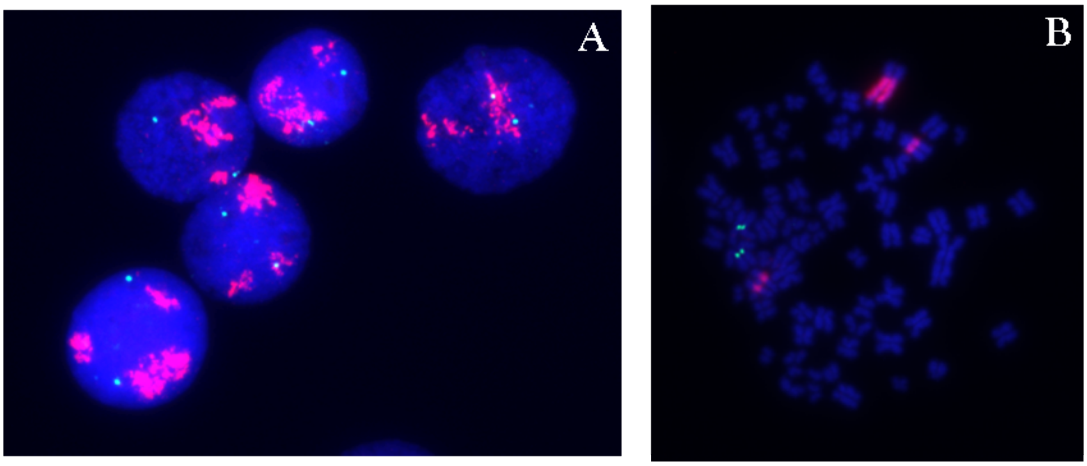

Figure S2-5. *MYCN* FISH in BE(2)C cells. Cells with 2 chromosomes 2 and *MYCN* amplification appear in interphase (A) and in metaphase (B). Specifically, in metaphase FISH, 2 chromosomes with centromere 2 are observed, in which the *MYCN* signals are barely seen, masked by the intensity of the amplifications. On the other hand, there are 3 other derived chromosomes that contain an amplification of *MYCN* in the form of homogeneously stained regions, but without a centromere of 2. Green: centromere of chromosome 2; Red: *MYCN*.

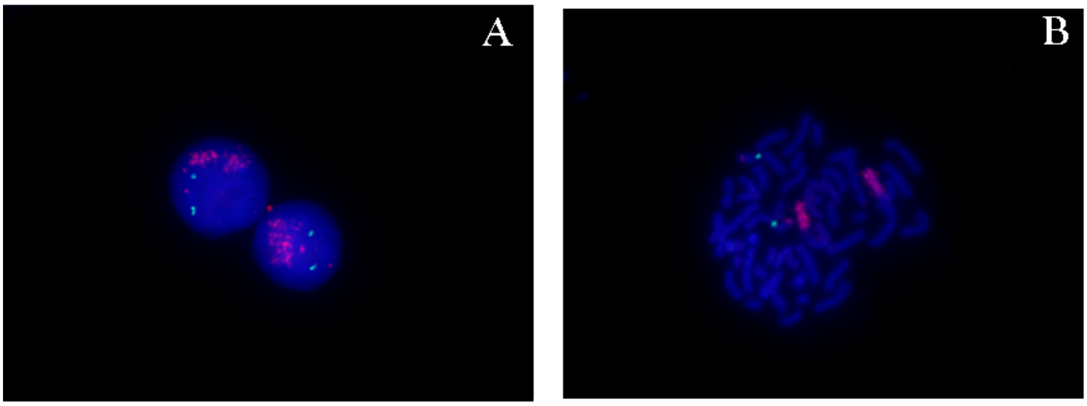

Figure S2-6. ***MYCN*** **FISH in IMR-32 cells.** FISH analysis was carried out on cell extensions in interphase (A) and metaphase (B). In the metaphase extensions, 2 chromosomes with centromere 2 and the respective *MYCN* signals are clearly distinguished. Therefore, there is colocalization of the signals in these chromosomes (chromosomes 2 "normal"). In addition, *MYCN* probe analysis revealed a characteristic homogeneously stained regions shaped gene amplification signal distribution on 2 other derived chromosomes of unknown origin, without chromosome 2 centromere. Green: chromosome 2 centromere; Red: *MYCN*.

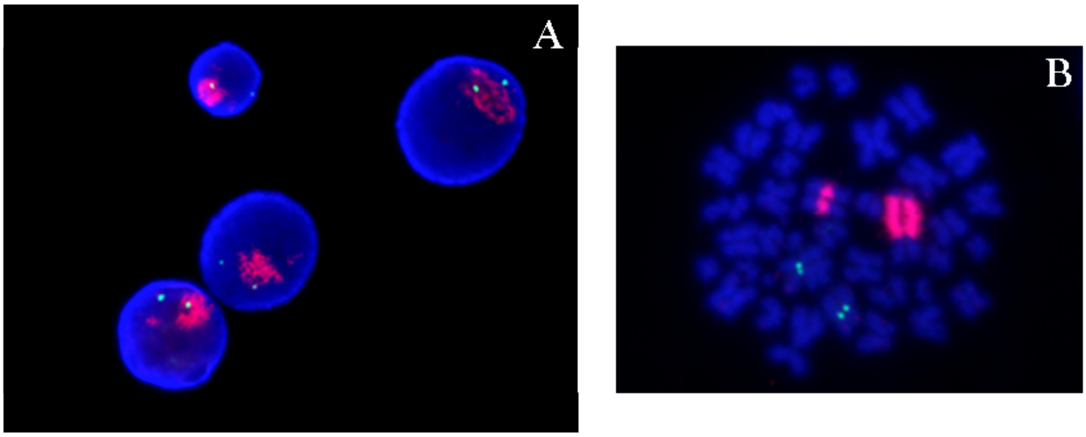

Figure S2-7. *MYCN* FISH in SK-N-Be(2) cells. FISH analysis was carried out on cell extensions at interphase (A) and metaphase (B). Two chromosomes with centromere 2 and the respective *MYCN* signals were detected in metaphases, despite not being able to stand out well in the photographs. They are therefore "normal" chromosomes 2. Two other derived chromosomes of unknown origin were also observed showing *MYCN* amplification in homogeneously stained regions but without the centromere of chromosome 2. Polyploid metaphases were seen in the preparations. Green: centromere of chromosome 2; Red: *MYCN*.

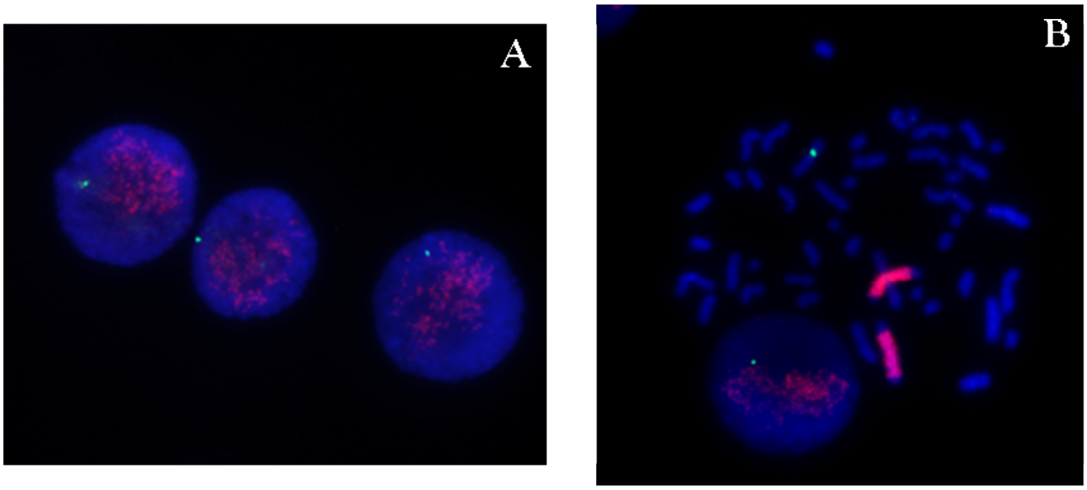

Figure S2-8. *MYCN* FISH in SK-N-FI cells. Cell nuclei show a pattern of an α2 signal in green and *MYCN* amplification in red, either in interphase (A) or metaphase (B) extensions. Green: centromere of chromosome 2; Red: *MYCN*.

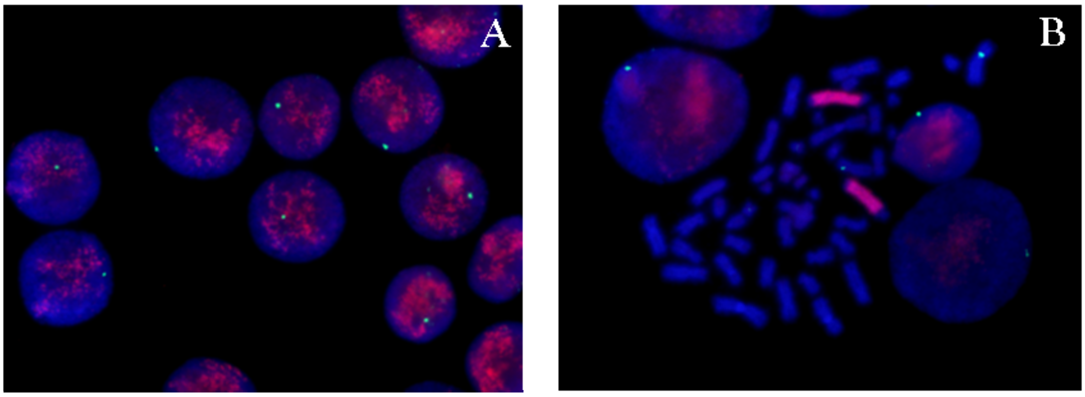

Figure S2-9. ***MYCN*** **FISH in Kelly cells.** The only clone of this cell line shows a hybridization pattern of a green signal of the α2 probe (chromosome 2 centromere) and amplification of the signal of RP11-744F11 (containing the *MYCN* gene), marked in red, in interphase cell extensions (A) and in metaphases (B). Green: centromere of chromosome 2; Red: *MYCN*.

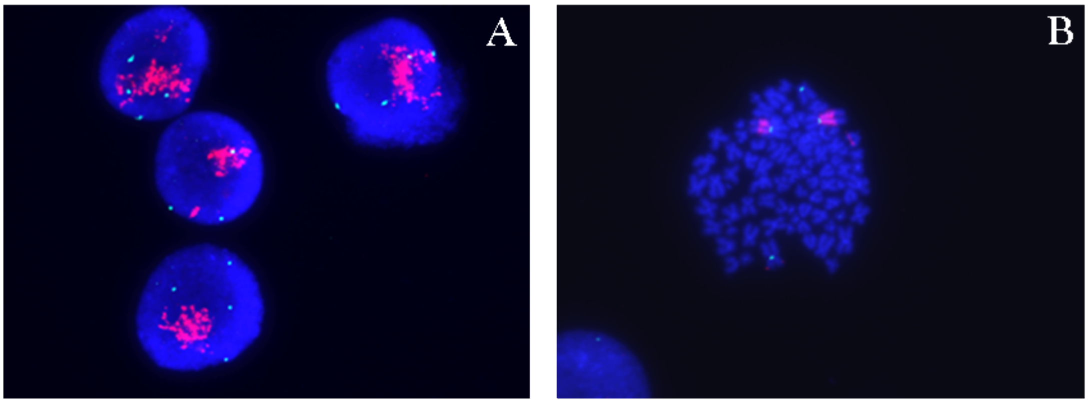

Figure S2-10. *MYCN* FISH in SK-N-DZ cells. These cells presented *MYCN* amplification in interphase (A) and metaphase (B) preparations. In metaphase FISH, 4 chromosomes with centromere 2 were observed: two of them contained *MYCN* amplification in homogeneously stained regions, another one showed loss of *MYCN*, and the last one presented a normal hybridization pattern. In addition, there is another chromosome without a centromere of chromosome 2 and with *MYCN* amplification in the form of a homogeneously stained region. Green: centromere of chromosome 2; Red: *MYCN*.

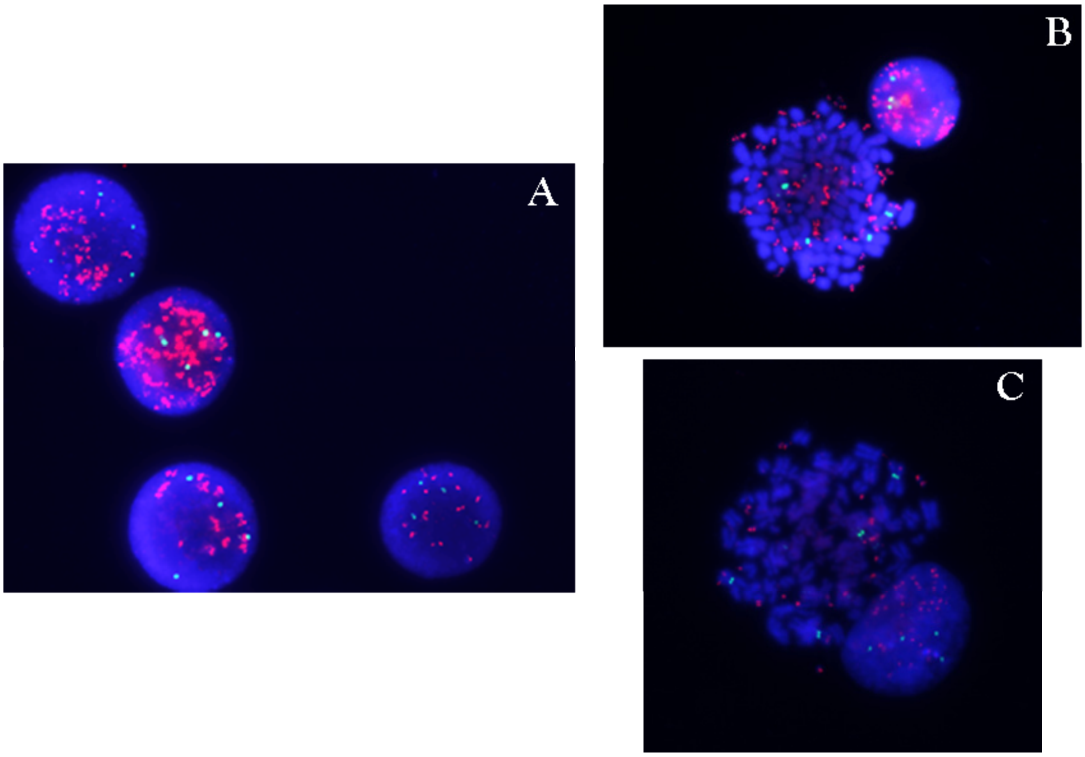

Figure S2-11. ***MYCN*** **FISH in SIMA cells.** FISH analysis was carried out on cell extensions in interphase (A) and metaphase (B,C). The analysis carried out indicated the existence of cells with 4 chromosomes 2 and a very large amplification of *MYCN* (> 20 copies) or with a lower degree of amplification of the gene (< 20 copies), a difference that can be clearly seen in the photograph of the nuclei in interphase (A), showing a diffuse amplification pattern for *MYCN*. In the metaphase extensions (B,C), *MYCN* red signals were observed both inside and outside the chromosomes, compatible with double minute chromosomes . Green: centromere of chromosome 2; Red: *MYCN*.

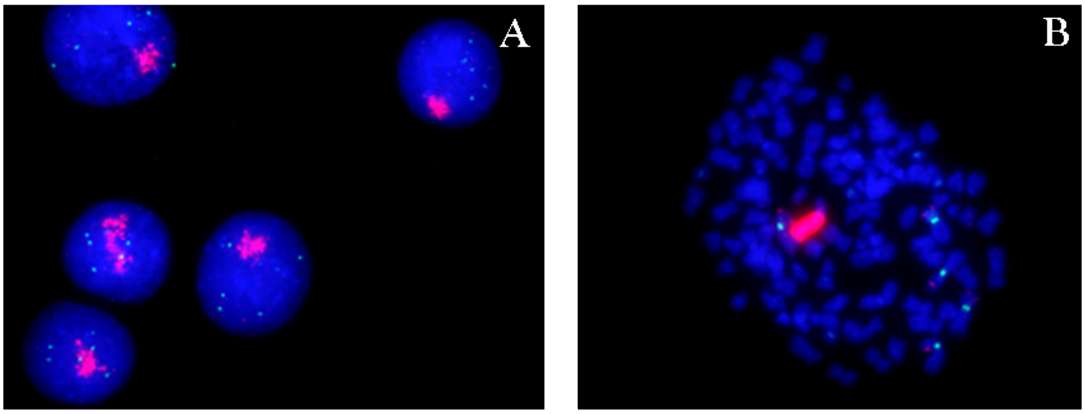

Figure S2-12. *MYCN* FISH in MHH-NB-11 cells. FISH analysis was carried out on cell extensions at interphase (A) and metaphase (B). Multiple copies of *MYCN* were detected in the interphase extensions (A). In metaphase (B), colocalization of green and red signals are found on 5 chromosomes and a chromosome of unknown origin that presented a homogeneously stained region amplified *MYCN*. Green: centromere of chromosome 2; Red: *MYCN*.

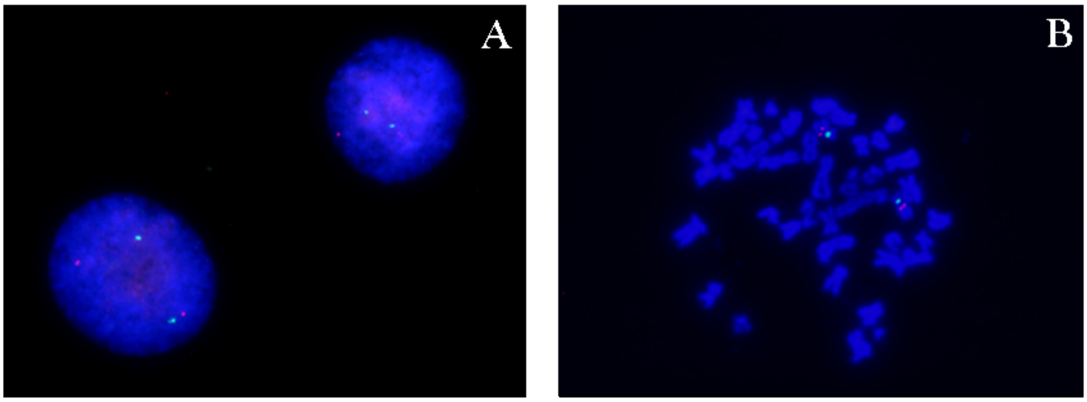

Figure S2-13. ***MDM2*** **FISH in SK-N-MC cells.** Normal hybridization pattern in interphase (A) and metaphase (B) cell extensions is shown. Green: centromere of chromosome 12; Red: *MDM2*.

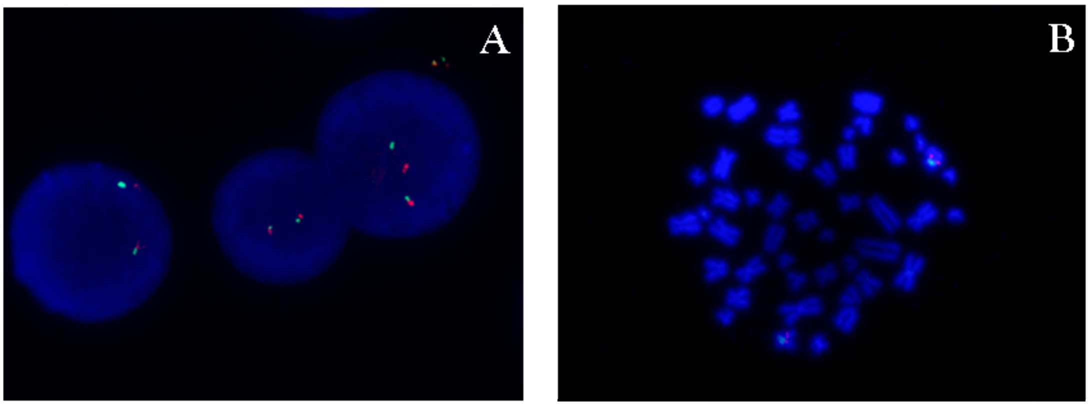

Figure S2-14. ***MDM2*** **FISH in SK-N-FI cells.** Normal hybridization pattern obtained in interphase (A) and metaphase (B) cell extensions is shown. The metaphase image indicates colocalization of green and red signals on the same chromosome. Green: centromere of chromosome 12; Red: *MDM2*.

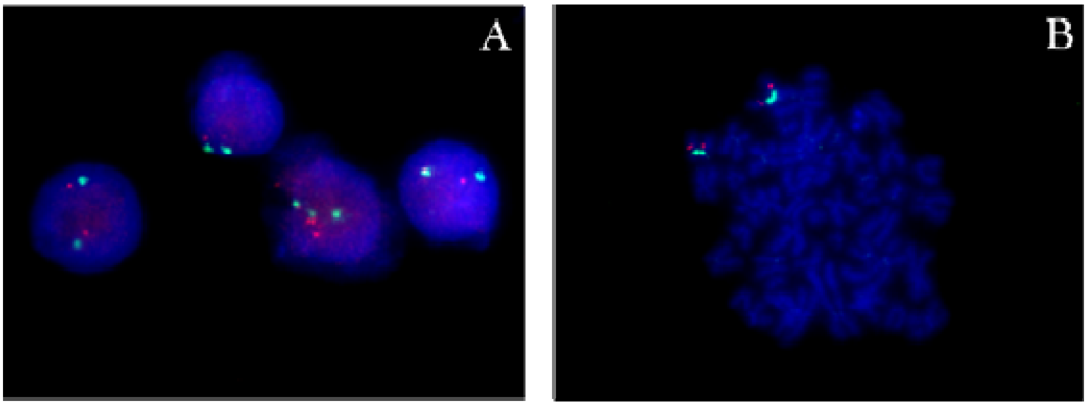

Figure S2-15. ***MDM2*** **FISH in IMR-32 cells.** Normal hybridization pattern found in these cells in interphase (A) and in metaphase (B) is shown. Green: centromere of chromosome 12; Red: *MDM2*.

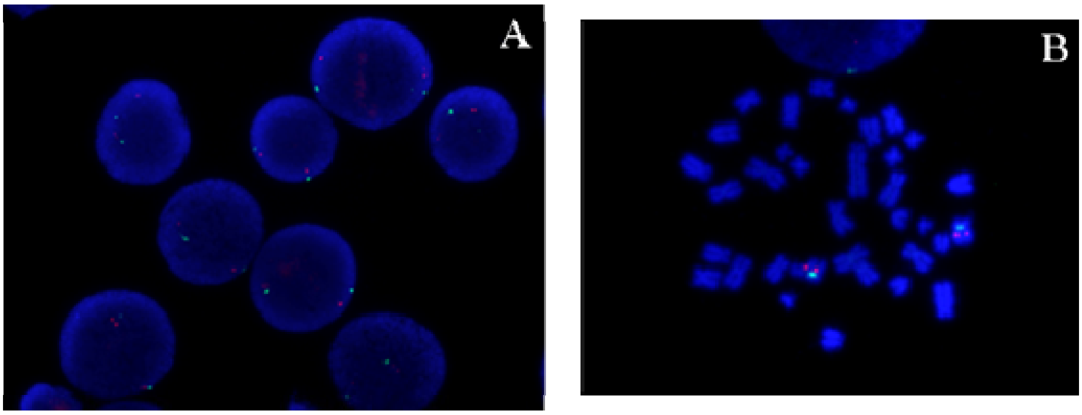

Figure S2-16. ***MDM2*** **FISH in Kelly cells.** All nuclei of the cells analyzed gave a 2Green 2Red count in both interphace (A) and metaphase (B). Green: centromere of chromosome 12; Red: *MDM2.*

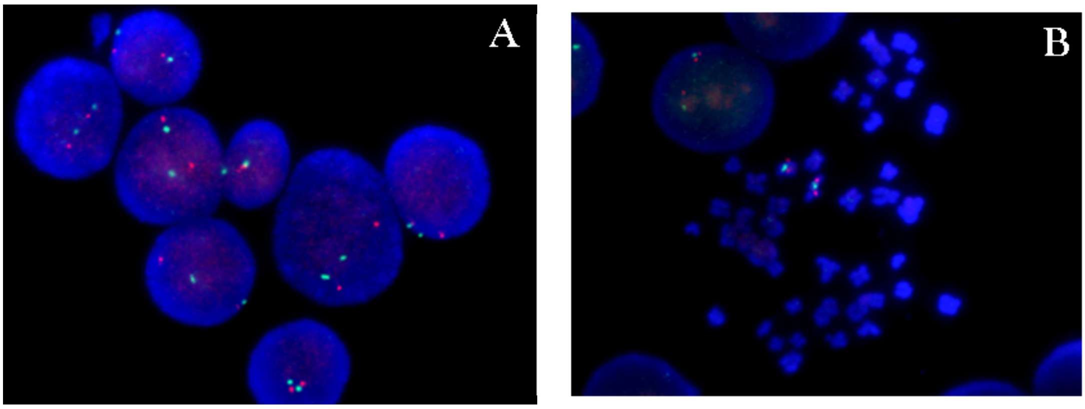

Figure S2-17. ***MDM2*** **FISH in SK-N-Be(2) cells.** The major clone in these cells presents two signals corresponding to the centromere of chromosome 12 and 2 signals from RP11-77H17 (which contains the *MDM2* gene) in interphase (A) and in metaphase (B). Green: centromere of chromosome 12; Red: *MDM2*.

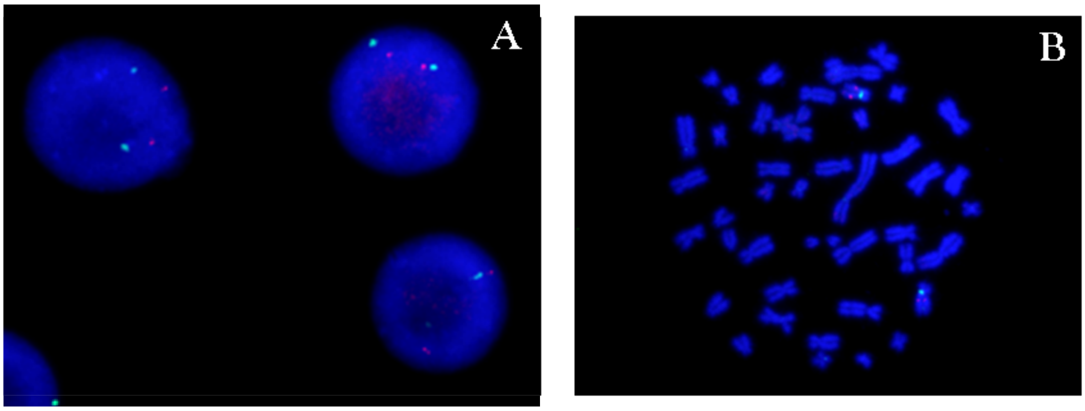

Figure S2-18. ***MDM2*** **FISH in SH-SY5Y cells.** All nuclei analyzed revealed, both in interphase (A) and in metaphase (B), a normal hybridization pattern. Green: centromere of chromosome 12; Red: *MDM2*.

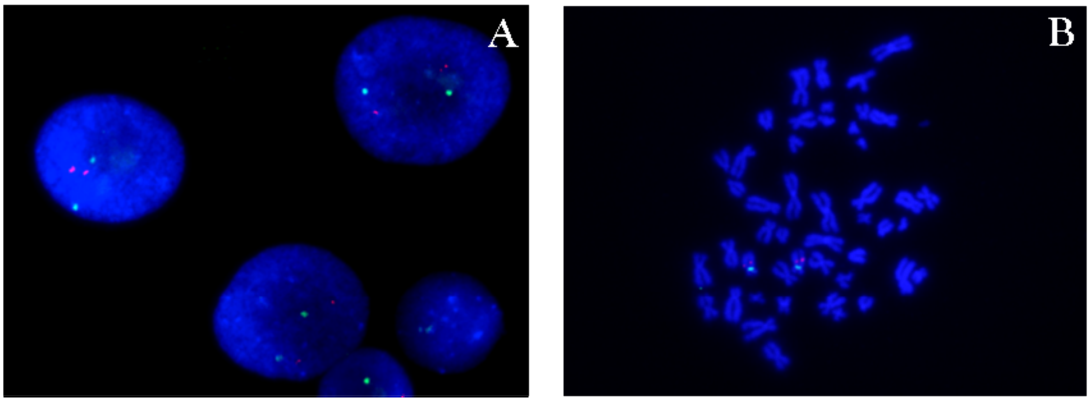

Figure S2-19. ***MDM2*** **FISH in SK-N-SH cells.** All nuclei analyzed revealed, both in interphase (A) and in metaphase (B), colocalization of signals corresponding to the centromere of chromosome 12 and to the *MDM2* gene. Green: centromere of chromosome 12; Red: *MDM2*.

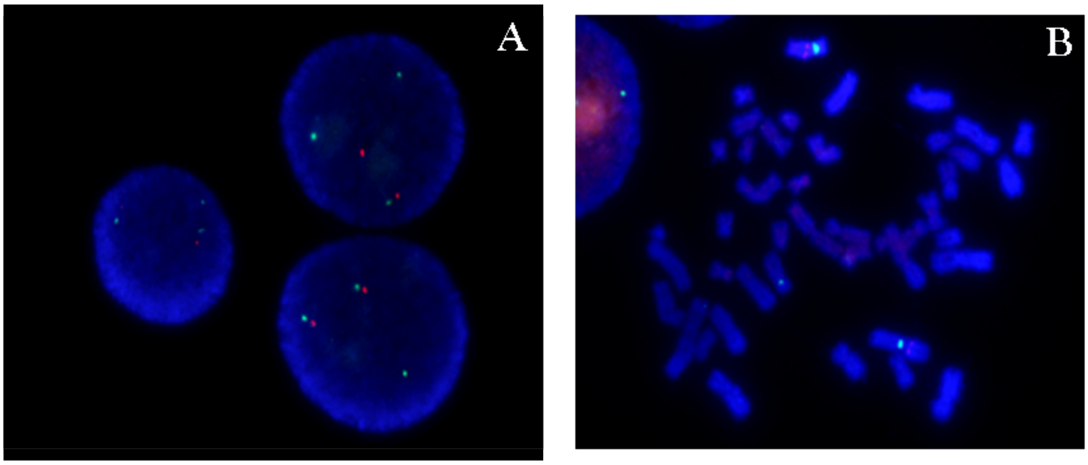

Figure S2-20. ***MDM2*** **FISH in MC-IXC cells.** Loss of *MDM2* in both interphase (A) and metaphase (B) cells. Green: centromere of chromosome 12; Red: *MDM2*.

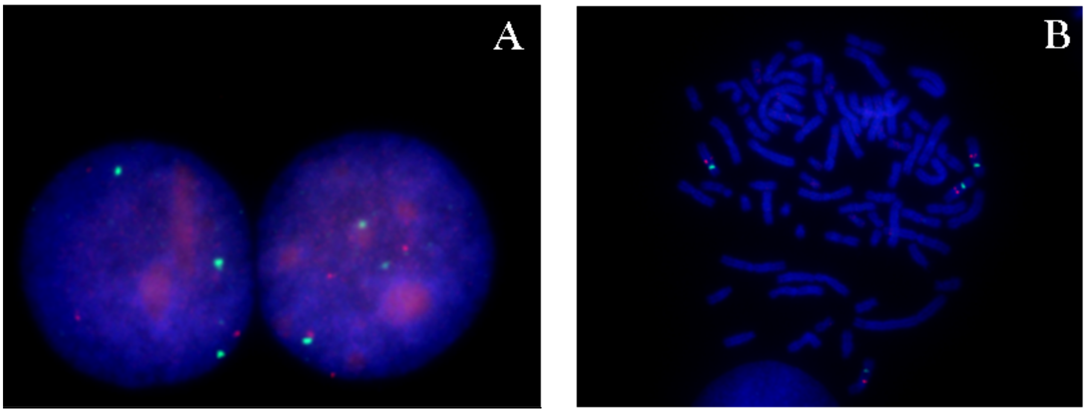

Figure S2-21. ***MDM2*** **FISH in BE(2)C cells.** Most nuclei present 3 green signals from the centromere of chromosome 12 and 3 red signals corresponding to *MDM2* (3G 3R) in interphase (A) and in metaphase (B). Green: centromere of chromosome 12; Red: *MDM2*.

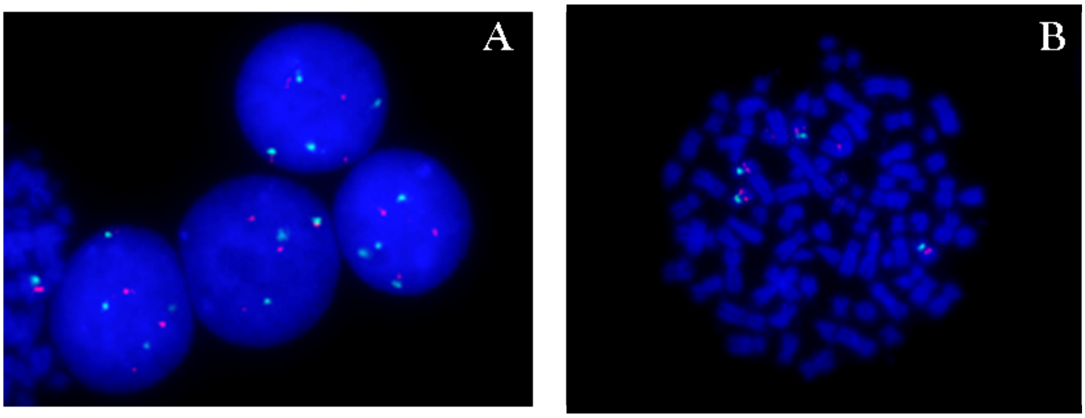

Figure S2-22. ***MDM2*** **FISH in SIMA cells.** Counting of the major clone of the cells, in interphase (A) and in metaphase (B), presented 4 signals of α12 and 4 signals of RP11-77H17 (4G 4R). Green: centromere of chromosome 12; Red: *MDM2*.

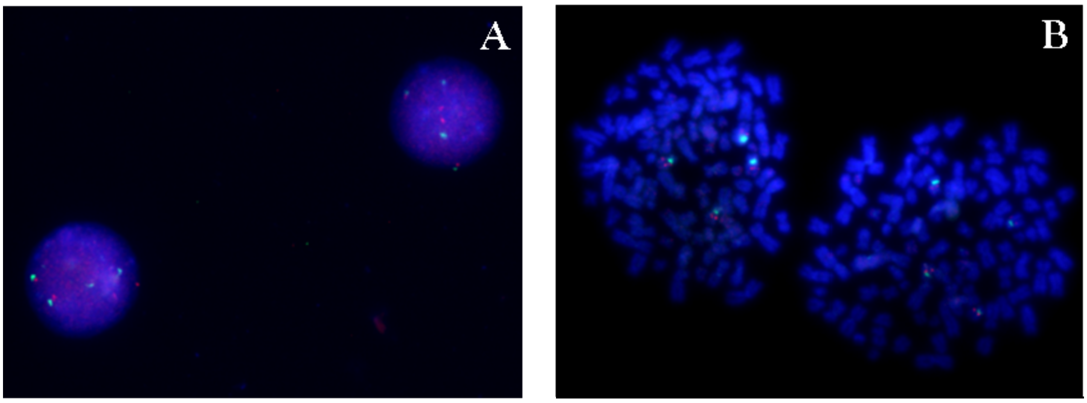

Figure S2-23. ***MDM2*** **FISH in MHH-NB-11 cells.** Images obtained from cell extensions in interphase (A) and in metaphase (B) are shown. The test showed a 4G 4R result, that is, 4 signals were detected from the centromere of the studied chromosome and another 4 signals from the *MDM2* gene, located therein. Green: centromere of chromosome 12; Red: *MDM2*.

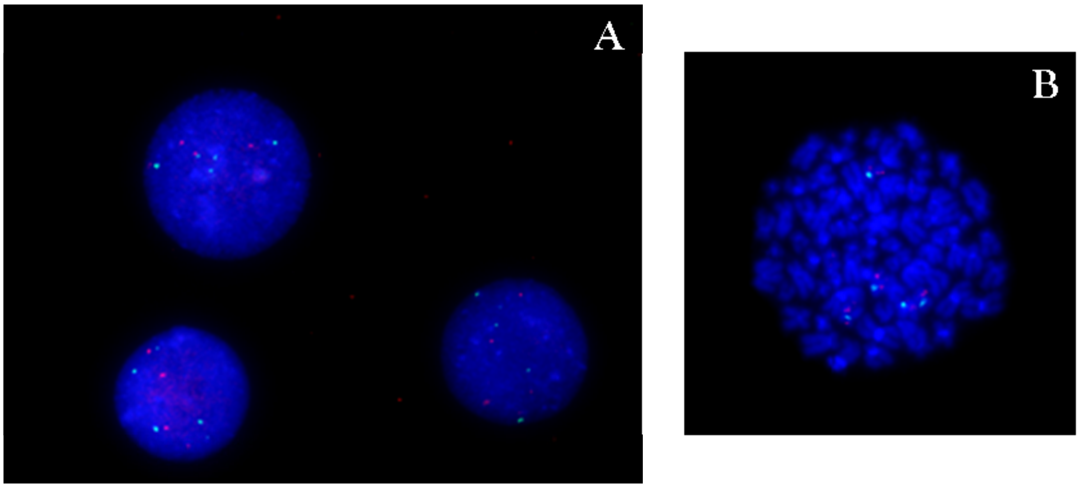

Figure S2-24. ***MDM2*** **FISH in SK-N-DZ cells.** In the interphase nuclei (A), 4 centromeres of chromosome 12, and a smaller one and 4 signals of the BAC that hybridizes with *MDM2* were observed (5G 4R). Metaphase analysis (B) confirmed that these α12 signals were cohybridized with *MDM2* on chromosome 12, except for one, which did not show *MDM2* hybridization in most nuclei. Green: centromere of chromosome 12; Red: *MDM2*.
