# Supplementary material for: In Vitro Assessment of the Role of p53 on Chemotherapy Treatments in Neuroblastoma Cell Lines
Source: Pharmaceuticals (Basel). 2021 Nov 19;14(11):1184. doi: 10.3390/ph14111184 (PMC8624165; doi:10.3390/ph14111184)
Supplement: Supplementary file 1 [file pharmaceuticals-14-01184-s001.zip › Figure S3.pdf]

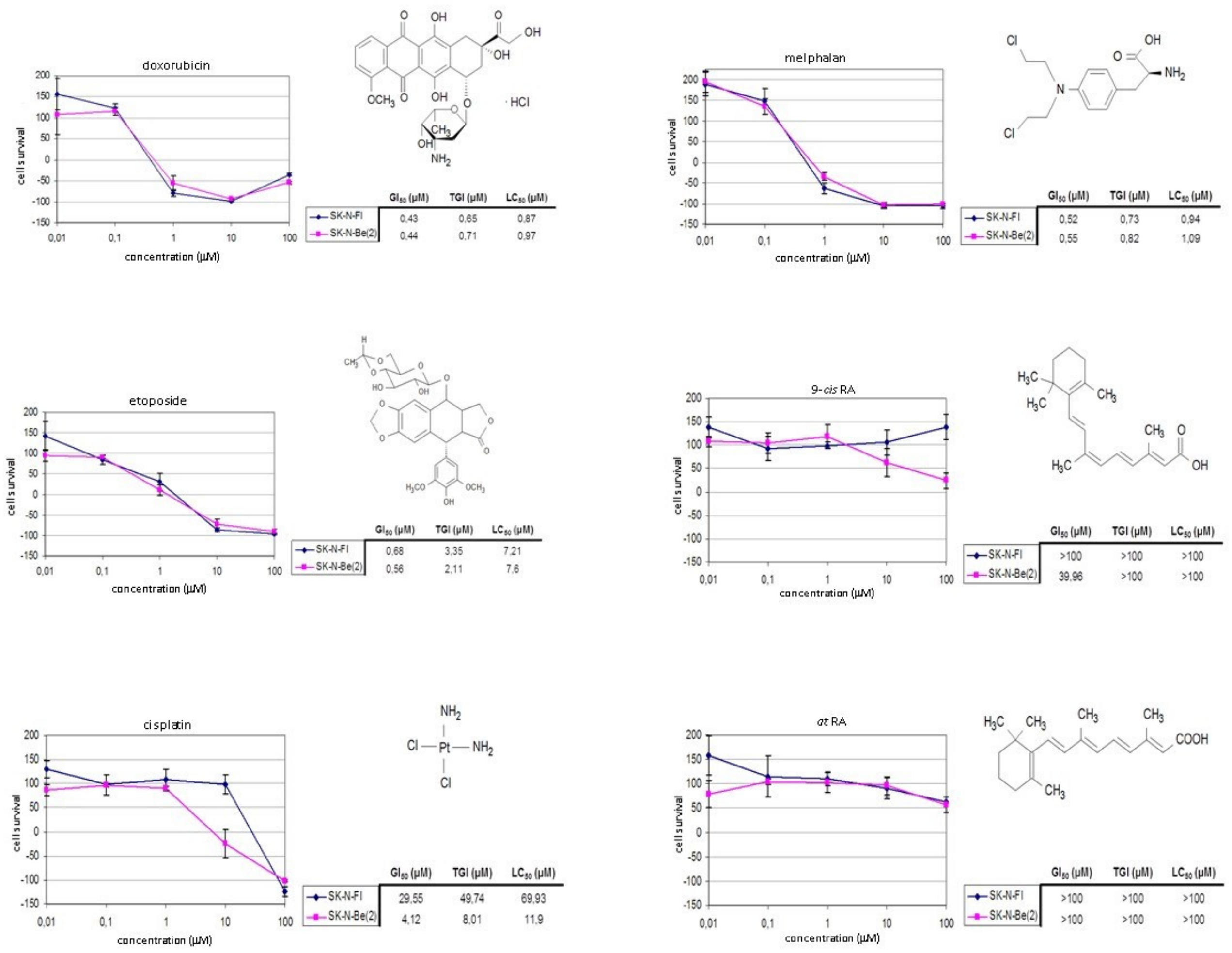

Figure S3. Analysis of cytotoxicity induced by doxorubicin, etoposide, cisplatin, melphalan, 9-*cis*-RA and *at*-RA in neuroblastoma cell lines SK–N–FI and SK–N–Be(2). The figure shows the dose-response curves obtained after 72 h of treatment. Data are presented as the mean ± SEM of at least 3 independent experiments.
