# Supplementary material for: In Vitro Assessment of the Role of p53 on Chemotherapy Treatments in Neuroblastoma Cell Lines
Source: Pharmaceuticals (Basel). 2021 Nov 19;14(11):1184. doi: 10.3390/ph14111184 (PMC8624165; doi:10.3390/ph14111184)
Supplement: Supplementary file 1 [file pharmaceuticals-14-01184-s001.zip › Supplementary Table S1.pdf]

Table S1-1. Status of the SK-NF-I cell line according to the IARC TP53 database (https://p53.iarc.fr/CellLines.aspx; accessed on 8 July 2021).

**Name**	**Topography**	**Morphology**	**ATCC**	**Cosmic**	**depmap**	**Sex**	**Age**	**Status**	**Exon**	**cDNA**	**Protein**	**PubMed**
SK-N-FI	NERVES	Neuroblastoma, NOS	CRL-2142	688087	ACH-000341	M	11	MUT	7-exon	c.737T>G	p.M246R	15188009

Table S1-2. Neuroblastoma cell lines with a mutated codon 135, as identified in the database.

**Name**	**Topography**	**Morphology**	**ATCC**	**Cosmic**	**depmap**	**Sex**	**Age**	**Status**	**Exon**	**cDNA**	**Protein**	**PubMed**
IMR/KAT100	NERVES	Neuroblastoma, NOS				NA		MUT	5-exon	c.404G>T	p.C135F	17974978
KP-N-YS	NERVES	Neuroblastoma, NOS		946363	ACH-002261	NA	4	MUT	5-exon	c.404G>T	p.C135F	15188009
SKNBE(2c)	NERVES	Neuroblastoma, NOS				NA		MUT	5-exon	c.404G>T	p.C135F	11196202
sknbe2	NERVES	Neuroblastoma, NOS			ACH-000312	M	2	MUT	5-exon	c.404G>T	p.C135F	22460905
SMS-BC	NERVES	Neuroblastoma, NOS				NA		MUT	5-exon	c.404G>T	p.C135F	9288759
UKF-NB-3rVCR	NERVES	Neuroblastoma, NOS				NA		MUT	5-exon	c.404G>T	p.C135F	17974978
